# Data mining the effects of testing conditions and specimen properties on brain biomechanics

**DOI:** 10.1080/23335432.2019.1621206

**Published:** 2019-06-03

**Authors:** Folly Patterson, Osama AbuOmar, Mike Jones, Keith Tansey, R.K. Prabhu

**Affiliations:** a Department of Agricultural and Biological Engineering, Mississippi State University, Starkville, MS, USA; b Center for Advanced Vehicular Systems, Mississippi State University, Starkville, MS, USA; c Department of Computing Sciences, Coastal Carolina University, Conway, SC, USA; d Department of Medical Engineering, Cardiff University, Cardiff, Wales, UK; e Department of Neurosurgery and Neurobiology, University of Mississippi Medical Center, Jackson, MS, USA; f Center for Neuroscience and Neurological Recovery, Methodist Rehabilitation Center, Jackson, MS, USA

**Keywords:** Traumatic brain injury, principal component analysis, fuzzy c-means clustering, self-organizing maps

## Abstract

Traumatic brain injury is highly prevalent in the United States. However, despite its frequency and significance, there is little understanding of how the brain responds during injurious loading. A confounding problem is that because testing conditions vary between assessment methods, brain biomechanics cannot be fully understood. Data mining techniques, which are commonly used to determine patterns in large datasets, were applied to discover how changes in testing conditions affect the mechanical response of the brain. Data at various strain rates were collected from published literature and sorted into datasets based on strain rate and tension vs. compression. Self-organizing maps were used to conduct a sensitivity analysis to rank the testing condition parameters by importance. Fuzzy C-means clustering was applied to determine if there were any patterns in the data. The parameter rankings and clustering for each dataset varied, indicating that the strain rate and type of deformation influence the role of these parameters in the datasets.

## Introduction

Traumatic brain injury (TBI) sent about 2.5 million people to the emergency room in the United States in 2013 (Taylor et al. 2017). Of these people, 56,000 died and 280,000 were hospitalized (Taylor et al. 2017). In Europe, approximately 2.5 million people will suffer a TBI each year; of these, 1 million will die and 75,000 will be hospitalized (Maas et al. 2015). TBI is most frequently caused by falls, blunt trauma, and motor vehicle accidents. TBI can cause a variety of long- and short-term health effects such as impaired memory, balance, and communication, as well as increased depression and anxiety. Furthermore, TBI increases the risk of Alzheimer’s disease and other neurological disorders. Approximately 5.3 million Americans live with a TBI-related disability. Such disabilities affect individuals’ relationships, productivity, and everyday living. The economic cost of TBI in the US was estimated to be $76.5 billion in 2010, with the vast majority of this amount coming from fatal TBIs and TBIs resulting in hospitalization. It is clear that TBI has a substantial impact on our society.

Understanding the biomechanics of TBI mechanisms is imperative if effective protective countermeasures are to be established. Numerous preclinical in vitro studies have been conducted in an attempt to improve understanding; however, the results of these studies vary in orders of magnitude in terms of the stress states applied to the brain material studied. This can be attributed to a number of reasons, such as in vitro specimen age (Chatelin et al. 2012), specimen storage and testing temperature (Zhang et al. 2011), specimen aspect ratio, and material heterogeneity (brain white [axons] or gray [neurons] matter, or a combination) (Prange et al. 2000; Pervin and Chen 2009, 2011; Chatelin et al. 2012). This is further compounded by significant inconsistencies in brain tissue biomechanical testing protocols. Though the ultimate goal of most of these tests is to obtain uniaxial stress-strain responses for brain tissue at quasi-static, intermediate, and high strain rates, the influence of the above factors on the data has not yet been quantified.

From quasi-static strain rates to high strain rates, brain tissue has been found to be highly strain-rate dependent (Miller and Chinzei 1997, 2002) as a result of its numerous structural components, which include solid and fluid materials. Brain tissue tends to be stiffer at higher loading rates (Miller and Chinzei 2002; Pervin and Chen 2009; Prabhu et al. 2011; Rashid et al. 2014). As a result, the peak stresses increase as strain rate increases, with the peak stresses varying across two orders of magnitude during quasi-static strain rate compression (Sparrey and Keaveny 2011).

Chatelin et al. (2012) observed that the stress response of the brain varies with the age of the individual from which specimens are taken, such that the adult human brain is 3–4 times stiffer than the infant brain. Thibault and Margulies (1998) concluded similarly that at low strains, the brains of 2–3 day old pigs were less stiff than one-year-old pigs. At large strains, Prange and Margulies (2002) found that immature pig brains were stiffer than adult pig brains.

A difference in the properties of brain white matter and gray matter has been reported extensively (Bilston et al. 1997; Ozawa et al. 2001; Van Dommelen et al. 2010); however, there is variation in the literature on the differences between white and gray matter. Prange et al. (2000) found that gray matter was on average stiffer than white matter by about 30% in porcine brain tissue. Nicolle et al. (2004) concluded gray matter was slightly stiffer than white matter, but concluded that both are similar enough at small strains. However, Manduca et al. (2001) found that white matter was three times stiffer than gray matter.

When under tension, brain tissue does not deform homogeneously because of the specimen edge effects where the brain specimen is attached to the apparatus platens. Larger diameter specimens undergo more inhomogeneous deformation than smaller diameter specimens (Rashid et al. 2012e); any variation in specimen diameter can have a large effect on stress response under tension. Under tension, a small specimen thickness is necessary for uniform deformation (Pervin and Chen 2011; Rashid et al. 2012e), but under compression, there are no significant differences in stress response at different thicknesses (Rashid et al. 2012c).

Gefen and Margulies (2004) consider post-mortem time to be the most important cause for variations in stress response in literature, though others have reached the opposite conclusion. Nicolle et al. (2004) concluded that differences in stress response in brain specimens tested between 24 and 48 h are insignificant. Zhang et al. (2011) found no change in response between two and six hours post-mortem and Prevost et al. (2011) found no response variations between four and 15 h. However, Sparrey and Keaveny (2011) found a change in the stress response porcine spinal cord white matter under compression despite all post-mortem preservation times being less than 4 h, and Garo et al. (2007) found that the thalamus increased in stiffness with increasing post-mortem time.

Tissue samples frequently must be stored for a few hours to a few days prior to testing. Generally, samples are stored at approximately 5°C, to minimize degradation effects which would affect the material response. Samples may also be stored at 37°C if testing will be performed within 4–6 h of extraction in order to mimic in vivo conditions. Zhang et al. (2011) studied the effect of storage temperature, ice-cold and 37°C, on the brain’s material response, finding that samples stored at 37°C exhibited a stiffer response than those stored at ice-cold temperature. Differences between the two responses decreased at higher strain levels. Zhang et al. (2011) concluded by recommending that researchers store brain tissue at low temperatures and perform tests at physiological temperatures. Brain tissue mechanical properties are also dependent on the temperature at which the samples are tested, as Hrapko et al. (2008) concluded that brain tissue is less stiff at room temperature (22°C-25°C) than at physiological temperature (37°C). There may also be more variability in testing temperature than reported, as the exact temperature of the room in which testing takes place is rarely measured.

In response to these unmet needs, unsupervised learning techniques were applied to determine how changes in brain mechanical properties relate to changes in testing conditions. One such technique, self-organizing maps, was used to conduct a sensitivity analysis on the data to determine which parameters were most significant. The principal component analysis was utilized to represent the data in a lower dimensional space. Finally, fuzzy C-means clustering with a Gustafson-Kessel distance measure was used to determine whether or not the datasets tend to cluster in certain patterns.

## Materials and methods

Experimental data were gathered from several brain tension and compression testing studies (Miller and Chinzei 1997, 2002; Shen et al. 2006; Tamura et al. 2008; Pervin and Chen 2009, 2011; Zhang et al. 2011; Rashid et al. 2012a, 2012b, 2012c, 2012d, 2012e, 2014; Li et al. 2015, 2019). The focus of this paper is on uniaxial tension and compression data. Studies on the shear, indentation, biaxial, etc. response of the brain were thus excluded. From this, 30 uniaxial tension and compression studies were found. Of these, those which used cylindrical brain samples were selected to remove the potential effect of geometry on the stress-strain response, leaving 15 studies. Several of these papers did not provide sufficient details that were essential for the analysis. Although there are techniques for analyzing data with missing values, the authors wanted to ensure there were as many characterizable relationships between the testing parameters and the mechanical responses of the brain as possible. A plot digitizer software tool developed by Ankit Rohatgi (2016) was used to extract the whole stress–strain curve from each published plot of these 15 studies. All parameters were converted to Systéme Internationale (SI) units and stress and strain were converted to true stress and strain, as required, for consistency. The data taken from each source are summarized in Table 1.

**Table 1.** Summary of data sources and associated parameters.

Age (mo)	Strain Rate	Testing	Specimen dimensions (mm)	Brain Matter Composition	Testing Temperature (°C)	Storage Temperature (°C)	Post-mortem Preservation Time (h)	Number of Data Points	Reference
6	Quasi-static	Compression	30×13	Gray	5	22	12	111	Miller and Chinzei (1997)
18	Quasi-static High	Compression	4.7×1.7	White Gray	37	25	4	996	Pervin and Chen (2009)
6 12 18	Quasi-static High	Compression	4.7×1.7	White Gray	37	25	4	1900	Pervin and Chen (2011)
6	High	Compression	13×3	Mixed	0 37	37	4	133	Zhang et al. (2011)
6	Intermediate	Compression	15×5	Mixed	5	22	8	219	Rashid et al. (2012c)
6	Quasi-static	Tension	30×10	Gray	5	22	12	185	Miller and Chinzei (2002)
6	Quasi-static	Compression	24×9	Mixed	4	37	24	85	Shen et al. (2006)
6	Intermediate	Tension	15×10	Mixed	5	22	4	116	Rashid et al. (2014)
6	Intermediate	Tension	15×415×715×10	Mixed	4	22	3	151	Rashid et al. (2012e)
6	Quasi-static Intermediate	Tension	14×14	White	4	22	4	190	Tamura et al. (2008)
6	Quasi-static Intermediate	Tension	15×315×415×515×615×8	Mixed	4.5	22	16.5	261	Rashid et al. (2012a)
6	Intermediate	Compression	15×6.1	Mixed	4.5	22	3	204	Rashid et al. (2012b)
						37			
6	Quasi-static Intermediate	Compression	15×5.1	Mixed	4.5	22	3	170	Rashid et al. (2012d)
1	Quasi-static Intermediate	Compression	9×5	Mixed	4.5	22	6	158	Li et al. (2015)
2	Quasi-static Intermediate	Compression	8×5	White Mixed	4	25	7	700	Li et al. (2019)

In the previous studies listed above (Miller and Chinzei 1997, 2002; Shen et al. 2006; Tamura et al. 2008; Pervin and Chen 2009, 2011; Zhang et al. 2011; Rashid et al. 2012a, 2012b, 2012c, 2012d, 2012e, 2014; Li et al. 2015, 2019), tension and compression, biomechanical tests were performed on brain tissue in order to characterize its deformation. In the case of compression, a small specimen of brain matter was placed between the top and bottom loading plates in the testing apparatus and compressed uniaxially at a specific constant displacement rate while the force and displacement, or strain, were measured. The brain specimens were cylindrical and typically cut out of the brain with a cylindrical die. A physiologically conducive solution, such as phosphate-buffered saline (PBS), can be used to immerse specimens during transportation and testing to prevent loss of moisture, which might affect the specimen’s stress response (Budday et al. 2015).

The input parameters of interest were: age of the individual from which specimens were taken, specimen diameter and thickness, specimen storage temperature prior to testing, specimen mechanical testing temperature, post-mortem preservation time, and brain matter composition. The species difference parameter was excluded because prior work has shown that there is no significant difference in brain properties between species (Pervin and Chen 2011). Brain matter composition was a categorical variable and has therefore been represented numerically. Strain rate was also recorded. Because the strain rate is ascertained by the researcher, it was considered an input parameter. Stress and strain were considered the output parameters of the dataset. Thus, the final dataset included eight input parameters and two output responses, with 5,579 data points. This dataset was then split into seven data subsets: tension, compression, quasi-static strain rate tension, intermediate strain rate tension, quasi-static strain rate compression, intermediate strain rate compression, and high strain rate compression. The data were normalized to the peak value of the corresponding variable so that all values shown in figures are between zero and one. The parameters of each data subset are listed in Table 2.

**Table 2.** The parameters and number of data points of each data subset.

	Tension	Compression	Quasi-static Strain Rate Tension	Intermediate Strain Rate Tension	Quasi-static Strain Rate Compression	Intermediate Strain Rate Compression	High Strain Rate Compression
Age		X			X	X	X
Strain Rate	X	X	X	X	X	X	X
Diameter	X	X	X	X	X	X	X
Thickness	X	X	X	X	X	X	X
Brain Matter Composition	X	X	X	X	X	X	X
Storage Temperature	X	X	X	X	X	X	X
Testing Temperature		X			X	X	X
Post-mortem Preservation Time	X	X	X	X	X	X	
Number of Data Points	903	4675	370	533	1686	755	2234

## Theory and calculations

Unsupervised learning techniques were applied to discover the patterns and relationships between the input testing conditions and the biomechanical stress-stress response. Because the focus of this work was not to understand cause-effect or predict the response, supervised learning was not employed. The data mining procedures used here to identify patterns in the data were: (1) Self-organizing maps (SOM), used to conduct a sensitivity analysis on the data to determine which parameters are most significant; (2) Principal component analysis (PCA), used to reduce the dimensionality; and (3) Fuzzy C-means clustering (FCM), used to analyze dimensionally reduced data using FCM clustering.

### Self-organizing maps

A Kohonen map, or self-organizing map (SOM), is a type of artificial neural network useful for visualizing patterns in high-dimensional data in a two-dimensional (2-D) or three-dimensional (3-D) array (Kohonen 1988). The inputs for the SOM are the dimensions of the dataset to be analyzed. Each input element connects to each neuron (an information-processing unit) in the array through a weight vector; after training, the SOM will create a mapping between the input space and the 2-D neuron map. The nonlinear SOM mapping uses a technique such that vectors which are close together in the higher dimensional space are also close together on the map.

SOM training is usually conducted on a 2-D neuron array with spatially defined neighborhoods, along with a method of data compression that determines the similarity of data. The SOM performs data compression such that the data is more convenient to handle with no loss of its complexity during compression. Using spatial neighborhoods allows for determining the similarity between the input vector and the vector of weights between the inputs and neurons.

Prior to training, weights are chosen randomly and an initial learning rate and neighborhood size are chosen. When a training vector comes in, the neuron with the closest weight is found, and the winning neuron’s weights are adjusted to make them even closer to the training input vector. This is repeated until convergence, when the feature map does not noticeably change between iterations. Once the artificial neural network (ANN) is properly initiated, there are three essential processes involved in the formation of the map (Kohonen 1988):

- *Competition*: for each input pattern, the neurons in the network compute their respective values of a discriminant function. This discriminant function provides the basis for competition among the neurons. The particular neuron with the largest value of discriminant function is declared the winner of the competition and this is the criterion for the winning neuron.
- *Cooperation*: the winning neuron determines the spatial location of a topological neighborhood of excited neurons, thereby providing the basis for cooperation among such neighboring neurons.
- *Adaption*: the synaptic weights of the winning neuron and its neighbors are adjusted such that their individual values of the discriminant function in relation to similar input patterns will be decreased.

In these datasets, the features are those listed in Table 2 and SOMs were produced with respect to each of these features. The feature(s) that has/have the most clustering tendency (i.e. there are groups or clusters of similar values in the SOM) is the most significant (important) in the dataset. If the clustering tendency is less dominant (i.e. data clusters don’t contain similar values), this means that the corresponding feature is less important. One way to know the exact order of significance is to produce an SOM with respect to one feature (say strain), then run the SOM but this time after removing one feature from the dataset (say diameter). If the clusters of the previous SOM (the one that was produced with respect to strain rate) remain the same as the case before removing ‘diameter’ from the dataset, this means that ‘diameter’ is not significant. However, if the structure of SOM changes after removing the feature ‘diameter’ this means that this particular feature is significant.

### Principal component analysis

It is difficult to visually represent and analyze a dataset’s patterns in high-dimensional space, in which there are more variables than can be easily visualized or analyzed using traditional statistical methods. As such, a technique like principal component analysis (PCA) can be used to determine patterns in the data and represent it in an easier to comprehend format by reducing the number of dimensions without losing the underlying data structure. Since different clustering techniques involve using a distance measure (e.g. Euclidean, Gustafson-Kessel, Manhattan, etc.) in order to assign different data vectors into the appropriate cluster, reducing dimensionality is important in order to calculate the distance matrix that can be used as the basis for building the membership matrix. However, clustering algorithms by themselves don’t reduce the dimensionality of data. Therefore, PCA was used to reduce the dimensionality to three dimensions in order to make it easier for fuzzy C-means clustering to work and calculate the corresponding distance and membership matrices.

The procedure for PCA was: (1) Calculate the mean across each parameter; (2) Subtract this mean from each parameter; (3) Find the covariance matrix and its eigenvectors and eigenvalues; and (4) Determine the principal components making up the dimensionally reduced datasets using the eigenvectors and eigenvalues. The number of principal components for each dataset was chosen such that the amount of variability in the data accounted for by the principal components was at least 85%.

### Fuzzy C-means clustering

After dimensionality reduction with PCA, the fuzzy C-means (FCM) clustering algorithm (Bezdek and Ehrlich 1984) was applied to find patterns in the stress-strain data. Clustering tends to involve a C×N membership matrix U, where C is the number of clusters and N is the number of data points. Each element in U represents the degree of membership of a datapoint to a cluster:

(3) $$U=\left[\begin{array}{c} u_{11}\\ \begin{array}{c} u_{21}\\ \vdots \\ u_{C1} \end{array} \end{array}\;\begin{array}{c} u_{12}\\ \begin{array}{c} u_{22}\\ \vdots \\ u_{C2} \end{array} \end{array}\;\begin{array}{c} \;\ldots \;\\ \begin{array}{c} \;\ldots \;\\ \ddots \\ \;\ldots \; \end{array} \end{array}\;\begin{array}{c} u_{1N}\\ \begin{array}{c} u_{21}\\ \vdots \\ u_{CN} \end{array} \end{array}\right]$$

For a hard partitioning of the stress state data into C clusters, each membership must be zero or one. Clustering can be achieved by optimizing a cost function, and then iteratively alternating estimates of the vectors in the cost function.

FCM is then an objective function-based clustering method, where $V=\left\{v_{1},\;\ldots \;,v_{C}\right\}$ with the initial value $v_{i}$ being the prototype for cluster i, set randomly, and

(4) $$\sum_{i=1}^{C}u_{ik}=1,\;\forall \;k=1,\;\ldots \;,N,$$

Meaning the memberships of each data vector must sum to one. The cost function for FCM can be written as,

(5) $$J\left(U,V\right)=\sum_{i=1}^{C}\sum_{k=1}^{N}u_{ik}^{Q}d\left(x_{k},v_{i}\right),$$

where *Q* is the fuzzifier, or weighting exponent (1≤Q<∞), and $d\left(x_{k},v_{i}\right)$ is the distance metric between data vector $x_{k}$ and cluster center $v_{i}$. A Gustafson-Kessel distance measure (see Supplementary Material), scaled by a hyper-volume approximation, was used because it uses covariance matrices for each cluster, allowing the distance measure to capture the statistical features of each cluster.

Usually, the fuzzifier (*Q*) is chosen arbitrarily based on how soft or hard we want the partitioning. That is, large values of *Q* (≥4) result in softer partitioning. Hard partitioning means that the degree of membership of each data sample to a particular cluster is relatively high (roughly 0.7–1.0) whereas soft partitioning means that the degree of membership is lower (roughly 0.5–1.0). On the other hand, crisp partitioning is the toughest measure of hard partitioning where each data sample should have a degree of membership of ‘1’ to be assigned to a particular cluster. These degrees of membership are determined by our choice of the fuzzifier *Q*, so it is quite hard to come up with a mathematical formula or rationale to calculate *Q*. However, the choice of *Q* as 2 is a common practice as it is a midway between the crisp and soft cases (Bezdek and Ehrlich 1984).

## Results

### Self-organizing maps

**Figure 1.**
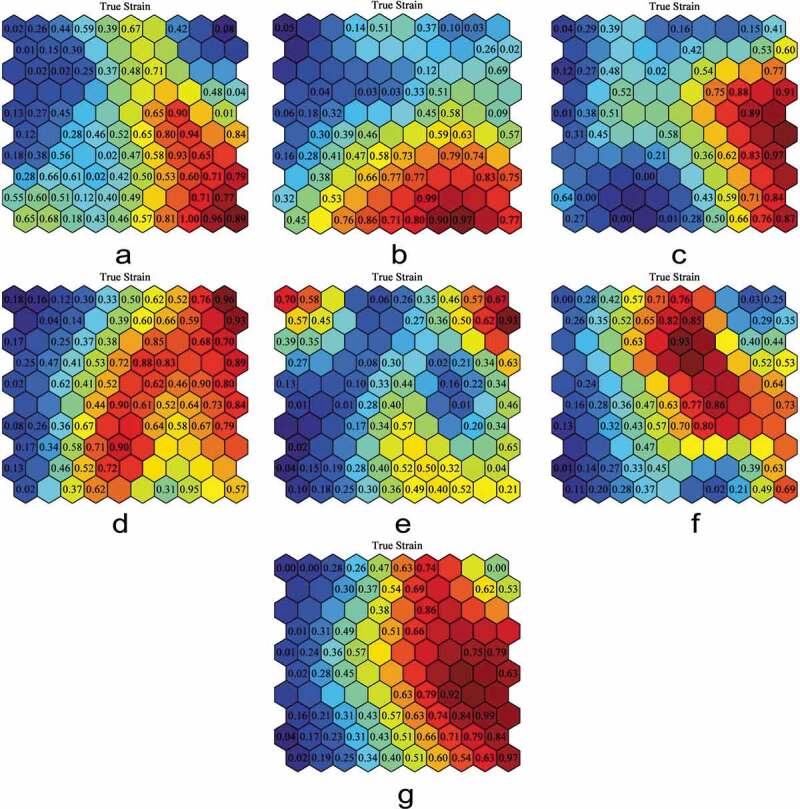
(a) 10 × 10 SOMs with respect to strain for: (a) Compression, (b) Quasi-static strain rate compression, (c) Intermediate strain rate compression, (d) High strain rate compression, (e) Tension, (f) Quasi-static strain rate tension, (g) Intermediate strain rate tension.

In Figure 1, the 10 × 10 SOMs with true strain labels are shown for the seven datasets, which have been used for comparison in determining the ranks of the input parameters. It is important to note that the values shown in the SOMs are normalized with the peak value of the corresponding testing parameter (strain, storing temperature, testing temperature, etc.). Brain matter composition, which is a categorical variable, is given the value 0.33 for white matter, 0.66 for gray matter, and 1.00 for mixed gray and white matter. The figures used to determine parameter ranking can be found in the Supplementary Material.

For the compression dataset, the parameters in order of significance are testing temperature, age, brain matter composition, diameter, strain rate, post-mortem preservation time, storage temperature, and thickness. The compression dataset has a similar sensitivity to testing temperature, age, and brain matter composition.

For quasi-static strain rate compression, the parameters in order of significance are storage temperature, age, testing temperature, strain rate, thickness, brain matter composition, post-mortem preservation time, and diameter.

The intermediate strain rate compression parameter rankings are: thickness, diameter and post-mortem preservation time (tied), testing temperature, storage temperature, brain matter composition, strain rate, and age. The strain was similarly sensitive to thickness, diameter, and post-mortem preservation time, indicating that these three parameters are of similar significance.

For high strain rate compression, the parameters in order of significance are age; brain matter composition; strain rate and age (tied); diameter, thickness, and testing temperature (tied); and storage temperature.

The parameters for tension data listed in order of significance are: post-mortem preservation time, strain rate and brain matter composition (tied), diameter, storage temperature, and thickness.

The parameter rankings for the quasi-static strain rate tension data in order are thickness, strain rate, post-mortem preservation time, storage temperature, brain matter composition, and diameter. For the intermediate strain rate tension data, the parameter rankings are brain matter composition and diameter (tied), thickness, storage temperature, strain rate, and post-mrotem preservation time. The SOM results are summarized in Table 3.

**Table 3.** Ranks of testing condition parameters and brain specimen properties in each of the seven data sets.

Rank	Tension	Compression	Quasi-static Strain Rate Tension	Intermediate Strain Rate Tension	Quasi-static Strain Rate Compression	Intermediate Strain Rate Compression	High Strain Rate Compression
1	Post-mortem Preservation Time	Testing Temperature	Thickness Strain Rate	Brain Matter Composition Age	Storage Temperature	Thickness	Brain Matter Composition
2	Strain Rate Brain Matter Composition	Age	Strain Rate	Thickness	Age	Diameter Post-mortem Preservation Time	Strain Rate
3	Diameter	Brain Matter Composition	Post-mortem Preservation Time	Storage Temperature	Testing Temperature	Testing Temperature	Age
4	Storage Temperature	Diameter	Storage Temperature	Strain Rate	Strain Rate	Storage Temperature	Diameter Thickness Testing Temperature
5	Thickness	Strain Rate	Brain Matter Composition	Post-mortem Preservation Time	Thickness	Brain Matter Composition	Storage Temperature
6	s	Post-mortem Preservation Time	Diameter		Brain Matter Composition	Age	
7		Storage Temperature			Post-mortem Preservation Time		
8		Thickness			Diameter		

### Fuzzy C-means clustering

**Figure 2.**
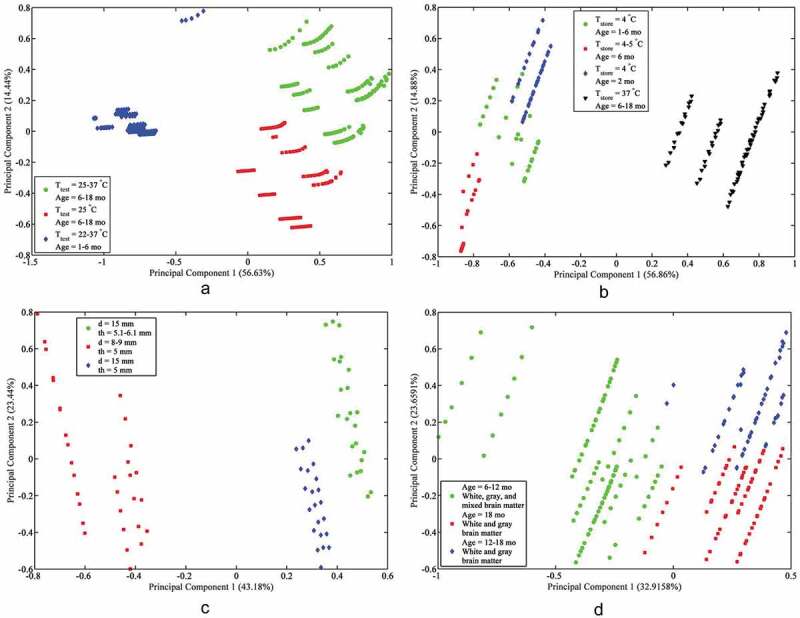
(a) Principal component analysis and fuzzy c-means clustering results for: (a) Compression, (b) Quasi-static strain rate compression, (c) Intermediate strain rate compression, (d) High strain rate compression.

Following PCA, FCM was run on the stress state data using a Gustafson-Kessel distance measure. The number of clusters for each dataset was chosen such that the maximum membership value for all or the majority of the data was over 0.5. The PCA and FCM plots for the compression data illustrated in Figure 2(a) show that it tends to cluster based on testing temperature and age. The quasi-static strain rate compression data have four clusters, as seen in Figure 2(b), based on storage temperature ad age. The intermediate strain rate compression data in Figure 2(c) cluster based on diameter and thickness The high strain rate compression data, shown in Figure 2(d), cluster according to brain matter composition and age. See the Supplementary Material for scaled image plots of cluster membership matrices.

**Figure 3.**
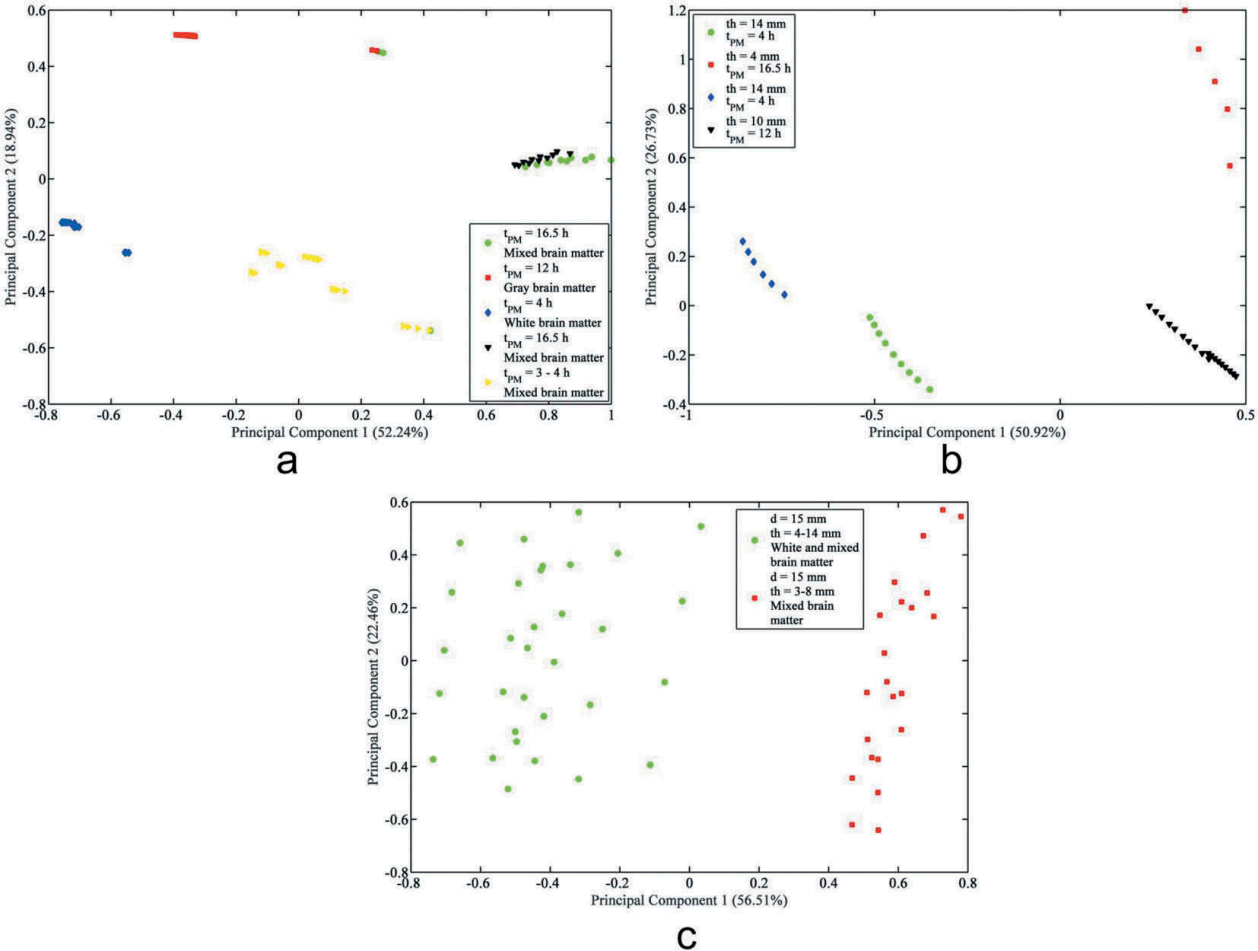
(a) Principal component analysis and fuzzy c-means clustering results for: (a) Tension, (b) Quasi-static strain rate tension, (c) Intermediate strain rate tension.

In Figure 3, the results of the PCA with FCM clustering for the tension, quasi-static strain rate tension, and intermediate strain rate tension datasets are shown. In Figure 3(a) the tension data form five clusters based on post-mortem preservation time and brain matter composition. In Figure 3(b), the quasi-static strain rate tension data has four clusters based on thickness and post-mortem preservation time. The intermediate strain rate tension data in Figure 3(c) forms two clusters based on brain matter composition and diameter.

## Discussion

The brain is a complex collection of tissues, with both heterogeneous and anisotropic regions. There are several studies which attempt to quantify and describe this behavior by deforming brain tissue under quasi-static, intermediate, and high strain rates, and under tension and compression (Miller and Chinzei 2002; Rashid et al. 2012a, 2014). However, the stress-strain responses and the conclusions thereof on brain tissue biomechanics are inconsistent due to the difficulty in building closed form solutions describing the data. The result is disparate data leading to inconsistent conclusions about brain tissue biomechanics. Data mining is an empirical approach which can explain potential sources of variation in the data and is undertaken here in order to find the trends in brain tissue biomechanical data and correlate them with in vitro testing conditions and brain specimen properties.

Three data mining techniques were utilized to analyze the data: self-organizing maps (SOM), fuzzy C-means clustering (FCM), and principal component analysis (PCA). Because these methods revealed several trends that were expected, such as strain rate and age dependencies, we concluded that the methods were appropriate for the problem at hand. The SOM analysis allows for the initial identification of potential groups in the data and reveals significant testing condition parameters. The FCM clustering method is used to identify clusters in order to accurately categorize the stress-strain data, as well as assign different levels of cluster membership to the data, or the degree to which each datapoint belongs to each cluster.

The SOMs in Figure 1 give the variations in clustering behavior between each dataset – compression, quasi-static compression, intermediate strain rate compression, high strain rate compression, tension, quasi-static strain rate tension, and intermediate strain rate tension. In other words, it can be inferred from the clustering pattern differences that there are significant variations due to changes in strain rate regimes (quasi-static, intermediate, and high) and stress state (compression and tension) on the brain tissue’s mechanical responses. The relevance of strain rate and stress state dependence has been documented in the body of literature (Miller and Chinzei 1997, 2002; Pervin and Chen 2009, 2011; Rashid et al. 2014). From Figure 1(b–d), due to the distinct variations in the cluster patterns, one can assert that strain rate plays a pivotal role in the stress-strain behavior of the brain parenchyma.

With regards to the datasets used here, strain rate ranked second in the tension dataset (Table 3), and was separated into the FCM clusters by quasi-static and intermediate rates (Figure 3(a)). Strain rate ranked second in the quasi-static strain rate tension data, again due to the strain rate dependency of brain tissue, though its significance was similar to the thickness and post-mortem preservation time. The four clusters of the quasi-static strain rate tension data each had significantly different strain rates (0.9 s^−1^ for cluster 1, 2 s^−1^ for cluster 2, 4.3 s^−1^ for cluster 3, and 0.0064–0.64 s^−1^ for cluster 4) (Figure 3(b)). Strain rate, however, ranked next to last in the intermediate strain rate tension data, and its clusters in Figure 3(c) had varying strain rates, indicating that the sensitivity of true strain to brain matter composition, diameter, and thickness are just as significant as the strain rate dependency of brain tissue. Hence, it is critical to include strain rate dependency and consider experimental specimen dimensions for the constitutive modeling of the brain under tensile deformation.

Strain rate also ranked second in the high strain rate compression data. However, strain rate ranked fourth out of eight parameters for quasi-static strain rate compression, and seventh out of eight parameters for intermediate strain rate compression. While the strain rate dependency is evident in the quasi-static and high strain rate compression data, a similar trend is not apparent for intermediate strain rate compression data. The specimens for quasi-static and high strain rate tests were conducted using standardized methods (Gray and Blumenthal 2000) for uniaxial compression tests. However, intermediate strain rate tests were performed using a novel test setups that combined the attributes of quasi-static and high strain rate compression test apparatus. The lack of strain rate significance for the intermediate strain rate data could be due to the uniqueness of the intermediate strain rate testing methods. Further investigation into the differences of the testing procedure for intermediate strain rates is warranted to understand this anomaly.

Although a difference in the stiffness of mature and immature brain tissue has been found at low strains (Thibault and Margulies 1998) and high strains (Prange and Margulies 2002), age ranked second in quasi-static strain rate compression dataset, and third in the high strain rate compression dataset, but it ranked last in the intermediate strain rate compression dataset. The decrease in the rank of age the intermediate strain rate compression data is likely due to the lack of variability in age within this dataset. The range of age in the compression and quasi-static strain rate compression data is 1–18 months, and the range is 6–18 months for high strain rate compression. The range of age in the intermediate strain rate compression data is 1–6 months, indicating that the material properties of brain tissue are similar at these ages. Past 6 months, the material properties of brain tissue change over time. In other words, due to the stiffness of brain tissue, age-dependent brain tissue moduli are also strain rate-dependent.

Although there is disagreement in the literature on the difference in the mechanical properties of gray and white matter, there is enough of a difference between the two for brain matter composition to be a significant property of brain specimens (Bilston et al. 1997; Prange et al. 2000; Ozawa et al. 2001; Manduca et al. 2001; Nicolle et al. 2004; Van Dommelen et al. 2010). Hence, brain matter composition ranked second in the tension data and first in the intermediate strain rate tension data, and factored into the FCM clustering behavior as well (Figure 3(a–b)). In quasi-static strain rate tension, brain matter composition ranked fifth. In the compression dataset, brain matter composition ranked third, but it ranked sixth in quasi-static strain rate compression and fifth in intermediate strain rate compression. At lower strain rates for tension and compression, strain is less sensitive to brain matter composition than to other testing condition parameters. Further, mixed brain matter composition may have a varied mechanical response at the interface between brain white and gray matter relative to within them.

The FCM results show that two of the five clusters in the tension data contained quasi-static strain rates, but the diameters are quite different, at 14 mm for the first cluster and 30 mm for the second cluster, suggesting that diameter has a more significant effect on the stress response of brain tissue under tension in general compared to other parameters (Figure 3(a)). In each of the quasi-static strain rate datasets, diameter ranked last. This may be because the specimens were cut with an optimal diameter for each deformation condition. The intermediate and high strain rate data were more sensitive to diameter, however, indicating that the specimen diameter must be controlled carefully by the researcher under intermediate and high strain rates.

Thickness ranked first for the intermediate strain rate compression and quasi-static strain rate tension data, but ranked middle or last for the remaining datasets. Thickness ranked last in compression, third in high strain rate compression, and fifth in quasi-static strain rate compression; this coincides with previous work on changing specimen thickness under compression (Rashid et al. 2012c). Thickness ranked last in tension, presumably because most specimens were 10.0 mm thick or greater, which may reduce the effect of inhomogeneous deformation under tension (Rashid et al. 2012e). However, thickness ranked first in quasi-static strain rate tension and second in intermediate strain rate tension. The quasi-static strain rate tension data had thicknesses of 10 mm or 14 mm, indicating a significant change in stress response with this relatively small change in specimen thickness. The thicknesses in the intermediate tension data ranged from 3 mm to 14 mm, corresponding with a significant change in brain tissue stiffness due to thickness (Rashid et al. 2012a).

Post-mortem preservation time ranked seventh in the quasi-static strain rate compression data and second in the intermediate strain rate compression data. The majority of specimens in the quasi-static strain rate compression data were stored for 7 h or less, while the intermediate strain rate compression data contained specimens stored for 3–8 h. Post-mortem preservation time ranked first in the tension dataset, likely because the specimens in these data were stored between 3–16.5 hours. Post-mortem preservation time ranked sixth in the compression data; this might be because its effect on stress response was overshadowed by testing temperature, age, and brain matter composition. Though there is disagreement in the literature on the effect of post-mortem preservation time on brain tissue stress response, the strain is sensitive to post-mortem preservation times over 7 h.

Specimens in the quasi-static strain rate compression dataset were relatively evenly split between those stored at 4–5°C and those stored at 37°C, which may explain the data were more sensitive to storage temperature, which ranked first. Storage temperature ranked fourth in intermediate compression because all specimens were stored at 4–5°C, and ranked last in the high strain rate compression data, likely because most specimens were tested at 37°C and because the effect of storage temperature on stress response is decreased at higher strain rates (Zhang et al. 2011). In the tension dataset, storage temperature ranked next to last because all specimens were stored in near-freezing temperatures (4–5°C), the optimal storage temperature for reducing post-mortem degradation effects (Zhang et al. 2011). In the compression dataset, the majority of specimens were stored at physiological temperature, with only a few stored at room or near-freezing temperatures, hence the data were not sensitive to storage temperature.

In the compression data, the testing temperature ranked first. The specimens included in the compression data were tested at 22°C, 25°C, or 37°C, with previous work indicating that the brain is stiffer at physiological temperature than at room temperature (Hrapko et al. 2008). Testing temperature ranked third in the quasi-static and intermediate strain rate compression datasets. Testing temperature ranked third (next to last) in the high strain rate compression data, as most specimens in this dataset were tested around room temperature. Overall, the most significant parameters were testing temperature, age, and brain matter composition.

Strain rate dependency, across all compression and tension data, was observed to play an influential role in the stress state of the brain biomechanical dataset (Figure 1); be it tension or compression, or quasi-static or high strain rate. Currently, experiments are conducted with apparatuses that may be load-, displacement-, or strain-controlled, which may lead to inconsistent strain rates and non-uniform stress distribution. It would be beneficial to investigate novel ways to ensure consistent strain rates and uniform stress distribution during experiments. The choice of brain specimen region and orientation is critical, as there is great variability in the mechanical properties throughout the brain. There are significant differences in the properties of brain tissue due to age (Chatelin et al. 2012). Testing temperature also played a critical role in the biomechanical response of the brain tissue. Physiological temperature is 37°C, but the testing temperature varied from 22°C to 37°C, and as such, the mechanical response of the brain also varied with temperature. Since these parameters have a substantial influence on the brain’s mechanical response, brain constitutive material models that are calibrated to these biomechanical data should include these dependencies. Hence, it is pertinent to develop brain constitutive models that are strain rate, temperature, and heterogeneity (white vs. gray matter) dependent. Additionally, the thermal process by which the specimens are preserved and tested needs to be accounted for in the constitutive modeling process of the brain.

## Conclusion

Applying the proposed clustering techniques, the wide-ranging applications of data mining have been demonstrated in the context of biomechanical engineering, specifically in the area of soft tissue in vitro testing. The results from these data mining techniques contribute to a greater understanding of brain tissue biomechanics, as well as provide insight into the accuracy of brain tissue models. Since mechanical testing conditions can vary greatly from study to study, the results from each may be difficult to compare and may cause confusion about what stresses the brain is truly experiencing during TBI. The analysis performed here allowed for comparison across studies to determine the most salient conditions of brain tissue testing but it cannot necessarily provide a transformation function to correct for experimental condition differences between two studies that would make them truly comparable. Future work will focus on developing a multiple regression model for the data to predict the brain’s material properties under specific conditions. Further, the relationships determined here can improve the computational modeling of TBI. Data analyses like these may help experimentalists develop more consistent TBI model testing or data collection procedures, so that different studies could be more easily compared which might help the field achieve faster progress in biomechanical injury analysis. It is anticipated that data mining and machine learning methods will have wider relevance to the biomedical research community.

## Supplementary Material

Supplemental Material

## References

[CIT0001] Bezdek JC, Ehrlich R. 1984 FCM: the fuzzy c-means clustering algorithm. Comp Geosci. 10:191–203.

[CIT0002] Bilston LE, Liu Z, Phan-Thien N. 1997 Linear viscoelastic properties of bovine brain tissue in shear. Biorheology. 34:377–385. 9640354 10.1016/s0006-355x(98)00022-5

[CIT0003] Budday S, Nay R, de Rooij R, Steinmann P, Wyrobek T, Ovaert TC, Kuhl E. 2015 Mechanical properties of gray and white matter brain tissue by indentation. J Mech Behav Biomed Mater. 46:318–330. 25819199 10.1016/j.jmbbm.2015.02.024 PMC4395547

[CIT0004] Chatelin S, Vappou J, Roth S, Raul JS, Willinger R 2012 Towards child versus adult brain mechanical properties. J Mech Behav Biomed Mater. 6:166–173. 22301186 10.1016/j.jmbbm.2011.09.013

[CIT0005] Garo A, Hrapko M, van Dommelen JAW, Peters GWM 2007 Towards a reliable characterization of the mechanical behavior of brain tissue: the effects of post-mortem time and sample preparation. Biorheology. 44(1):51–58. 17502689

[CIT0006] Gefen A, Margulies SS 2004 Are in vivo and in situ brain tissue mechanically similar? J Biomech. 37:1339–1352. 15275841 10.1016/j.jbiomech.2003.12.032

[CIT0007] Gray GT, Blumenthal WR 2000 Split-Hopkinson pressure bar testing of soft materials In: Howard K, Dana M, editors. ASM Handbook, Mechanical testing and evaluation. Vol. 8 Materials Park (OH): ASM International; p. 488–496.

[CIT0008] Hrapko M, van Dommelen JAW, Peters GWM, Wismans JSHM 2008 The influence of test conditions on characterization of the mechanical properties of brain tissue. J Biomech Eng. 130:03100031–03100310. 10.1115/1.2907746 18532852

[CIT0009] Kohonen T 1988 Self-organization and associative memory. Verlag Berlin Heidelberg: Springer.

[CIT0010] Li K, Zhao H, Liu W, Yin Z 2015 Material properties and constitutive modelling of infant porcine cerebellum tissue in tension at high strain rate. PLoS One. 10(4):e0123506. 25830545 10.1371/journal.pone.0123506 PMC4382295

[CIT0011] Li Z, Yang H, Wang G, Han X, Zhang S 2019 Compressive properties and constitutive modelling of different regions of 8-week-old pediatric porcine brain under large strain and wide strain rates. J Mech Behav Biomed Mater. 89:122–131. 30268868 10.1016/j.jmbbm.2018.09.010

[CIT0012] Maas AIR, Menon DK, Steyerberg EW, Citerio G, Lecky F, Manley GT, Hill S, Legrand V, Sorgner A 2015 Collaborative European NeuroTrauma effectiveness research in traumatic brain injury (CENTER-TBI): A prospective longitudinal observational study. NeuroSurg. 76(1):67–80. 10.1227/NEU.0000000000000575 25525693

[CIT0013] Manduca A, Oliphant TE, Dresner MA, Mahowald JL, Kruse SA, Amromin E, Felmlee JP, Greenleaf RL, Ehman RL 2001 Magnetic resonance elastography: non-invasive mapping of tissue elasticity. Med Image Anal. 5:237–254. 11731304 10.1016/s1361-8415(00)00039-6

[CIT0014] Miller K, Chinzei K 1997 Constitutive modelling of brain tissue: experiment and theory. J Biomech. 30(11–12):1115–1121. 9456379 10.1016/s0021-9290(97)00092-4

[CIT0015] Miller K, Chinzei K 2002 Mechanical properties of brain tissue in tension. J Biomech. 35(4):483–490. 11934417 10.1016/s0021-9290(01)00234-2

[CIT0016] Nicolle S, Lounis M, Willinger R 2004 Shear properties of brain tissue over a frequency range relevant for automotive impact situations: new experimental results. Stapp Car Crash J. 48:239–258. 17230269 10.4271/2004-22-0011

[CIT0017] Ozawa H, Matsumoto T, Ohashi T, Sato M, Kokubun S 2001 Comparison of spinal cord gray matter and white matter softness: measurement by pipette aspiration method. J Neurosurg. 95(2 supp):221–224. 11599840 10.3171/spi.2001.95.2.0221

[CIT0018] Pervin F, Chen W 2009 Dynamic mechanical response of bovine gray matter and white matter brain tissues under compression. J Biomech. 42(6):731–735. 19269640 10.1016/j.jbiomech.2009.01.023

[CIT0019] Pervin F, Chen W 2011 Effect of inter-species, gender, and breeding on the mechanical behavior of brain tissue. NeuroImage. 54(1):S98–S102. 20362684 10.1016/j.neuroimage.2010.03.077

[CIT0020] Prabhu R, Horstemeyer MF, Tucker MT, Marin EB, Bouvard JL, Sherburn JA, Liao J, Williams LN 2011 Couple experiment/finite element analysis on the mechanical response of porcine brain under high strain rates. J Mech Behav Biomed Mater. 4:1067–1080. 21783116 10.1016/j.jmbbm.2011.03.015

[CIT0021] Prange MT, Margulies SS 2002 Regional, directional, and age-dependent properties of the brain undergoing large deformation. J Biomech Eng. 124:244–252. 12002135 10.1115/1.1449907

[CIT0022] Prange MT, Meaney DF, Margulies SS 2000 Defining brain mechanical properties: effects of region, direction and species. Stapp Car Crash J. 44:205–213. 17458728 10.4271/2000-01-SC15

[CIT0023] Prevost TP, Balakrishnan A, Suresh S, Socrate S 2011 Biomechanics of brain tissue. Acta Biomater. 7:83–95. 20603231 10.1016/j.actbio.2010.06.035

[CIT0024] Rashid B, Destrade M, Gilchrist M 2012a A high rate tension device for characterizing brain tissue. J Sport Eng Technol. 226:170–176.

[CIT0025] Rashid B, Destrade M, Gilchrist M 2012b Temperature effects on brain tissue in compression. J Mech Behav Biomed Mater. 14:113–118. 23022565 10.1016/j.jmbbm.2012.04.005

[CIT0026] Rashid B, Destrade M, Gilchrist M 2012c Mechanical characterization of brain tissue in compression at dynamic strain rates. J Mech Behav Biomed Mater. 10:23–38. 22520416 10.1016/j.jmbbm.2012.01.022

[CIT0027] Rashid B, Destrade M, Gilchrist M 2012d Determination of friction coefficient in unconfined compression of brain tissue. J Mech Behav Biomed Mater. 14:163–171. 23026694 10.1016/j.jmbbm.2012.05.001

[CIT0028] Rashid B, Destrade M, Gilchrist M 2014 Mechanical characterization of brain tissue in tension at dynamic strain rates. J Mech Behav Biomed Mater. 33:43–54. 23127641 10.1016/j.jmbbm.2012.07.015

[CIT0029] Rashid B, Destrade M, Gilchrist MD 2012e Inhomogeneous deformation of brain tissue during tension tests. Comp Mater Sci. 64:295–300.

[CIT0030] Rohatgi A 2016 WebPlotDigitizer; [accessed 2016 1212]. http://arohatgi.info/WebPlotDigitizer/app/.

[CIT0031] Shen F, Tay TE, Li JZ, Nigen S, Lee PV, Chan HK 2006 Modified Bilston nonlinear viscoelastic model for finite element head injury studies. J Biomech Eng. 128(5):797–801. 16995770 10.1115/1.2264393

[CIT0032] Sparrey CJ, Keaveny TM 2011 Compression behaviour of porcine spinal cord white matter. J Biomech. 44(6):1078–1082. 21353225 10.1016/j.jbiomech.2011.01.035

[CIT0033] Tamura A, Hayashi S, Nagayama K, Matsumoto T 2008 Mechanical characterization of brain in high-rate extension. J Biomech Sci Eng. 3(2):263–274.

[CIT0034] Taylor CA, Bell JM, Breiding MJ, Xu L 2017 3 Traumatic brain injury–related emergency department visits, hospitalizations, and deaths — United States, 2007 and 2013. Surveill Summ. 66(9):1–16. 10.15585/mmwr.ss6609a1 PMC5829835 28301451

[CIT0035] Thibault KL, Margulies SS 1998 Age-dependent material properties of the porcine cerebrum: effect on pediatric inertial head injury criteria. J Biomech. 31:1119–1126. 9882044 10.1016/s0021-9290(98)00122-5

[CIT0036] Van Dommelen JAW, van der Sande TPJ, Hrapko M, Peters GWM 2010 Mechanical properties of brain tissue by indentation: interregional variation. J Mech Behav Biomed Mater. 3:158–166. 20129415 10.1016/j.jmbbm.2009.09.001

[CIT0037] Zhang J, Yogananda N, Pintar FA, Guan Y, Shender B, Paskoff G, Laud P 2011 Effects of tissue preservation temperature on high strain-rate material properties of brain. J Biomech. 44(3):391–396. 21055756 10.1016/j.jbiomech.2010.10.024

