# Supplementary material for: Data mining the effects of testing conditions and specimen properties on brain biomechanics
Source: Int Biomech. 2019 Jun 3;6(1):34–46. doi: 10.1080/23335432.2019.1621206 (PMC7857311; doi:10.1080/23335432.2019.1621206)
Supplement: Supplemental Material [file TBBE_A_1621206_SM3139.zip › FCrawford_etal_IntlBiomech_Final_SuppMat.docx]

# Supplementary Materials

## Appendix A

The following outlines the determination of the cluster centers. Taking the partial derivative of the Lagrangian of the cost function $J\left( U,V \right)$ with respect to a specific membership value $u_{rs}$, shows that

$u_{rs}=\frac{1}{\sum_{i=1}^{C} \left( \frac{d\left( \boldsymbol{x}_{\boldsymbol{s}},\boldsymbol{v}_{\boldsymbol{r}} \right)}{d\left( \boldsymbol{x}_{\boldsymbol{s}},\boldsymbol{v}_{\boldsymbol{i}} \right)} \right)^{\frac{1}{Q-1}}}$.

The Gustafson-Kessel distance measure is given by:

$d_{ik}=\sqrt{\left| \boldsymbol{\Sigma}_{\mathbf{i}} \right|^{\frac{1}{D}}(\left( \boldsymbol{x}_{\boldsymbol{k}}-\boldsymbol{v}_{\boldsymbol{i}} \right)^{T}\boldsymbol{\Sigma}_{\boldsymbol{i}}^{-1}\left( \boldsymbol{x}_{\boldsymbol{k}}-\boldsymbol{v}_{\boldsymbol{i}} \right))}$.

Where the distance is scaled by a hyper-volume approximation $\left| \boldsymbol{\Sigma}_{\mathbf{i}} \right|^{\frac{1}{D}}$, and $\boldsymbol{\Sigma}_{\boldsymbol{i}}$ is the covariance matrix of class *i*, then

$\frac{\partial d_{ik}^{2}}{\partial\boldsymbol{v}_{\boldsymbol{i}}}=-2\left| \boldsymbol{\Sigma}_{\boldsymbol{i}} \right|^{\frac{1}{D}}\boldsymbol{\Sigma}_{\boldsymbol{i}}^{-1}(\boldsymbol{x}_{\boldsymbol{k}}-\boldsymbol{v}_{\boldsymbol{i}})$.

Thus, the cluster center of class *i* is given by

$\boldsymbol{v}_{\boldsymbol{i}}=\frac{\sum_{k=1}^{N} u_{ik}^{Q}\boldsymbol{x}_{\boldsymbol{k}}}{\sum_{k=1}^{N} u_{ik}^{Q}}$.

## Appendix B

A trained SOM along with its response to a winning output neuron is shown in Supp. Fig. 1 (King et al. 2005). The SOMs are excited by the unknown data input vector or the original training data vector(s). Domain experts finally check the data associated with the winning neuron to discover new domain knowledge. [Supplementary Figure 1 near here.]

The SOM algorithm operates as follows: First, there is an associated parameter reference vector *w_i_* for every neuron *i* on the SOM and the initial values of *w_i_* (0) are randomly chosen. Then, an input vector *x* (*R^n^*) is presented concurrently to all neurons on the map. The smallest value of the Euclidean distances is the measure used to define the best neuron that matches the introduced input vector *x*. However, other distance measures can be explored as well.

For the *competition* process mentioned in the main text (Kohonen 1988), let an m-dimensional input pattern selected at random from the input space be denoted as:

$$\boldsymbol{x}=\left[ x_{1},x_{2},\ldots,x_{m} \right]^{T}$$

Let the weight vector of the *j*th output neuron be denoted by:

$$\boldsymbol{w}_{\boldsymbol{j}}=\left[ w_{j1},w_{j2},\ldots,w_{jm} \right]^{T}, j=1,2,\ldots,l$$

The value of the *j*th output neuron is:

$$y_{j}=\boldsymbol{w}_{\boldsymbol{j}}^{T}\boldsymbol{x}, j=1,2,\ldots,l$$

A matching criterion is used to generate the winner:

$$i\left( \boldsymbol{x} \right)=\max_{j} (\boldsymbol{w}_{\boldsymbol{j}}^{T}\boldsymbol{x})$$

Or

$$i\left( \boldsymbol{x} \right)=\min_{j} \left| \left| \boldsymbol{x-}\boldsymbol{w}_{\boldsymbol{j}} \right| \right|$$

As a result, the SOM may be efficient in clustering the data vectors using these measures (King et al. 2005). As the training progresses, the radius of the neighborhood *N_m_* decreases over time such that $N_{m\left( t_{1} \right)}>N_{m(t_{2})}1>N_{m\left( t_{3} \right)}>\ldots>N_{m(t_{n})}$, where $t_{1}<t_{2}<t_{3}<\ldots<t_{n}$. That is, at the beginning of training, the size and influence of the neighborhoods can be very large, but as the training process ends, that neighborhood may contain only the winning neuron. Once again, the learning rate is decreased with time in the SOM algorithm.

Supplementary Figures 2-8 display the SOMs with strain labels for each dataset, but with one of each of the input parameters removed. If removing one parameter did not significantly change the clustering tendency of the output response, then it was concluded that the input parameter is not significant.

[Supplementary Figure 2 near here.]

[Supplementary Figure 3 near here.]

[Supplementary Figure 4 near here.]

[Supplementary Figure 5 near here.]

[Supplementary Figure 6 near here.]

[Supplementary Figure 7 near here.]

[Supplementary Figure 8 near here.]

## Appendix C

The scaled images of cluster membership matrices are shown below.

[Supplementary Figure 9 near here.]

[Supplementary Figure 10 near here.]

# References

King R, Rosenberger A, Kanda L. 2005. Artificial neural networks and three-dimensional digital morphology: A pilot study. Folia Primatol, 76:303-324.

Kohonen T. Self-organization and associative memory. Verlag Berlin Heidelberg: Springer, 1988.
