# Supplementary figures and images for: Data mining the effects of testing conditions and specimen properties on brain biomechanics
Source: Int Biomech. 2019 Jun 3;6(1):34–46. doi: 10.1080/23335432.2019.1621206 (PMC7857311; doi:10.1080/23335432.2019.1621206)

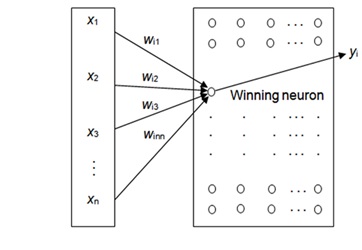

Supplement: Supplemental Material [file TBBE_A_1621206_SM3139.zip › Supplementary Figure 1.jpg]

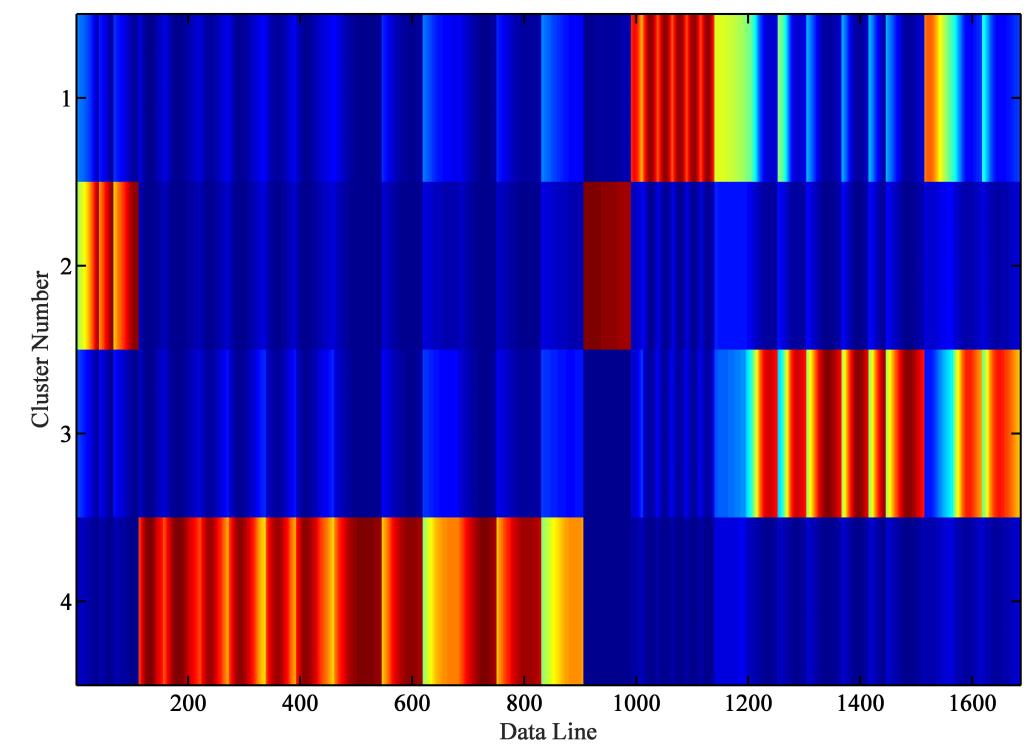

Supplement: Supplemental Material [file TBBE_A_1621206_SM3139.zip › Supplementary Figure 10.jpg]

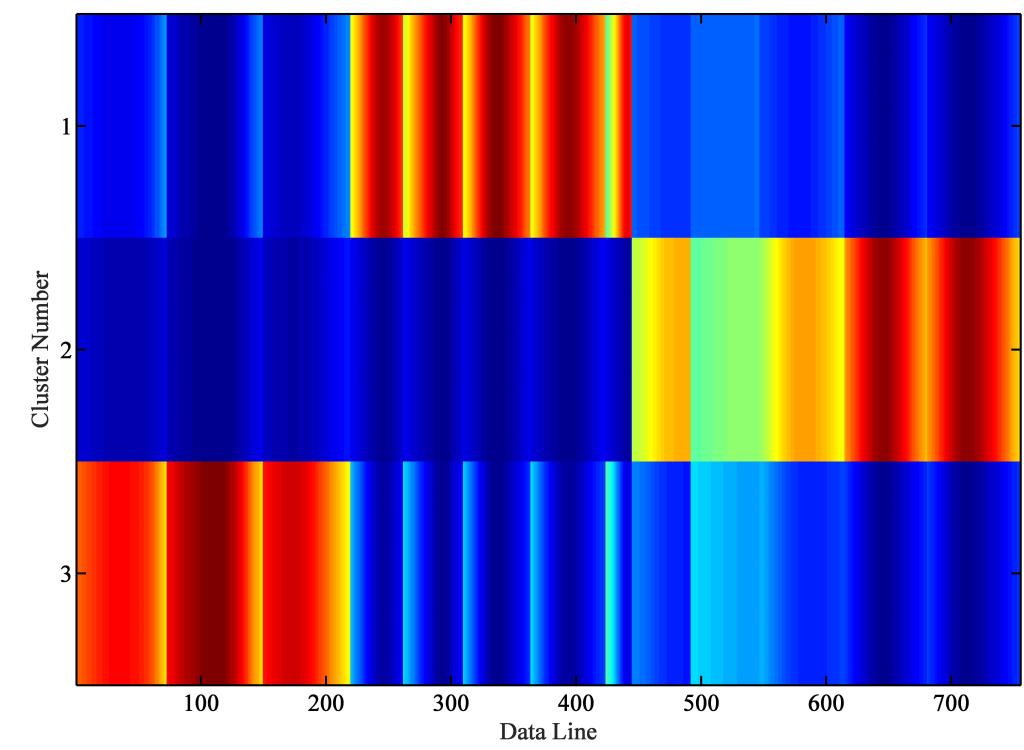

Supplement: Supplemental Material [file TBBE_A_1621206_SM3139.zip › Supplementary Figure 11.jpg]

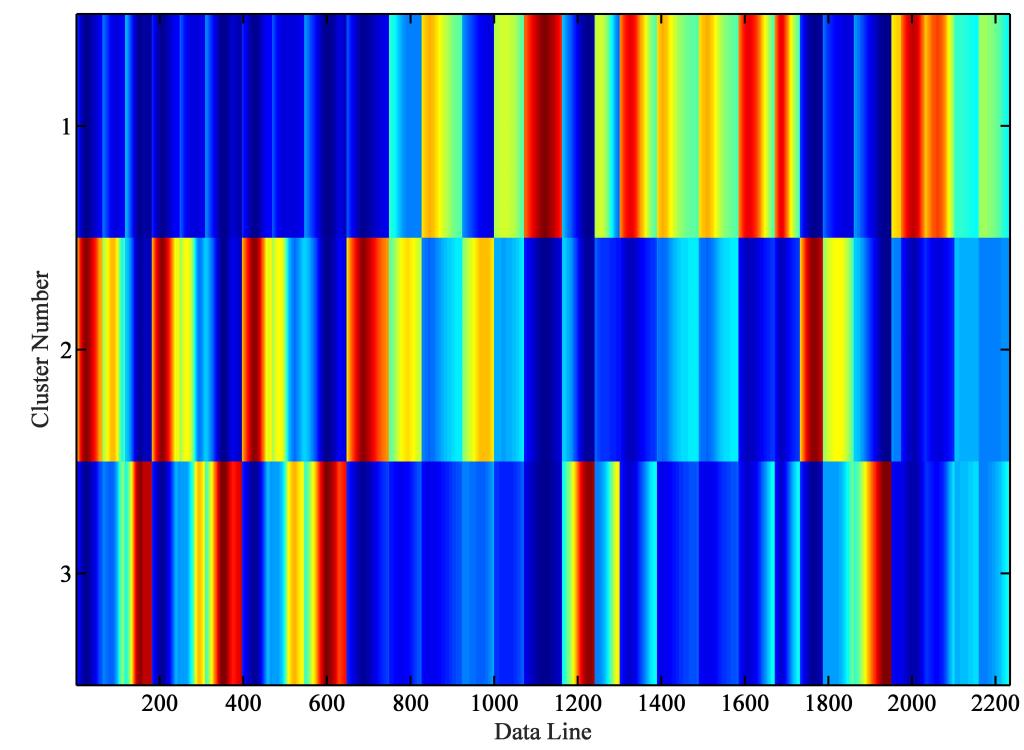

Supplement: Supplemental Material [file TBBE_A_1621206_SM3139.zip › Supplementary Figure 12.jpg]

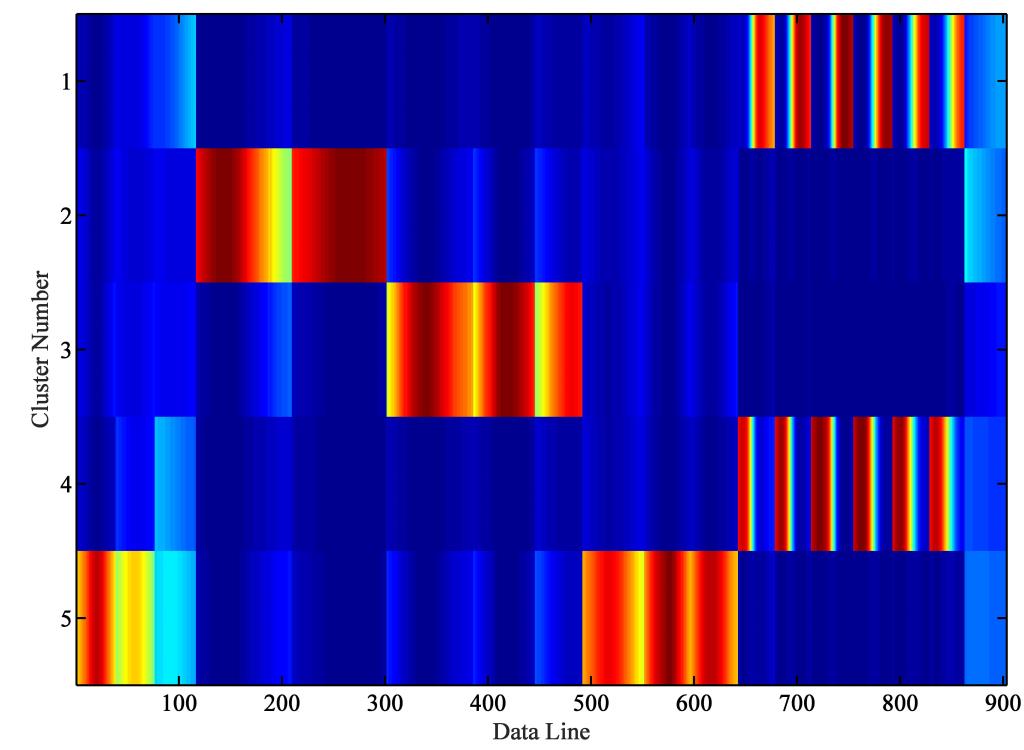

Supplement: Supplemental Material [file TBBE_A_1621206_SM3139.zip › Supplementary Figure 13.jpg]

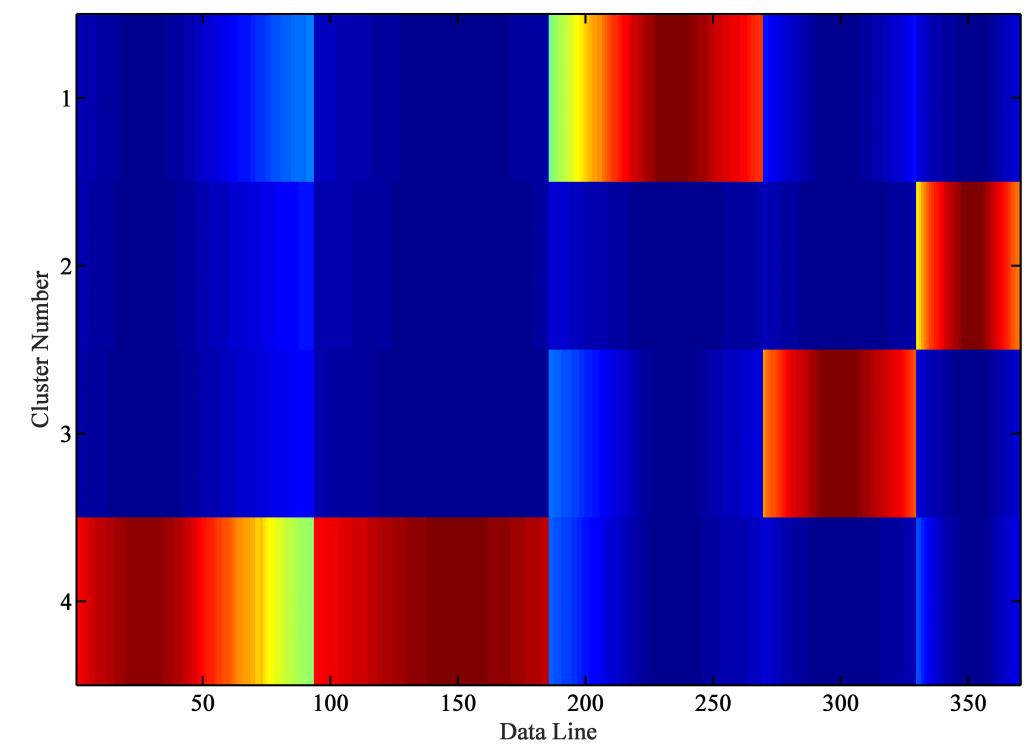

Supplement: Supplemental Material [file TBBE_A_1621206_SM3139.zip › Supplementary Figure 14.jpg]

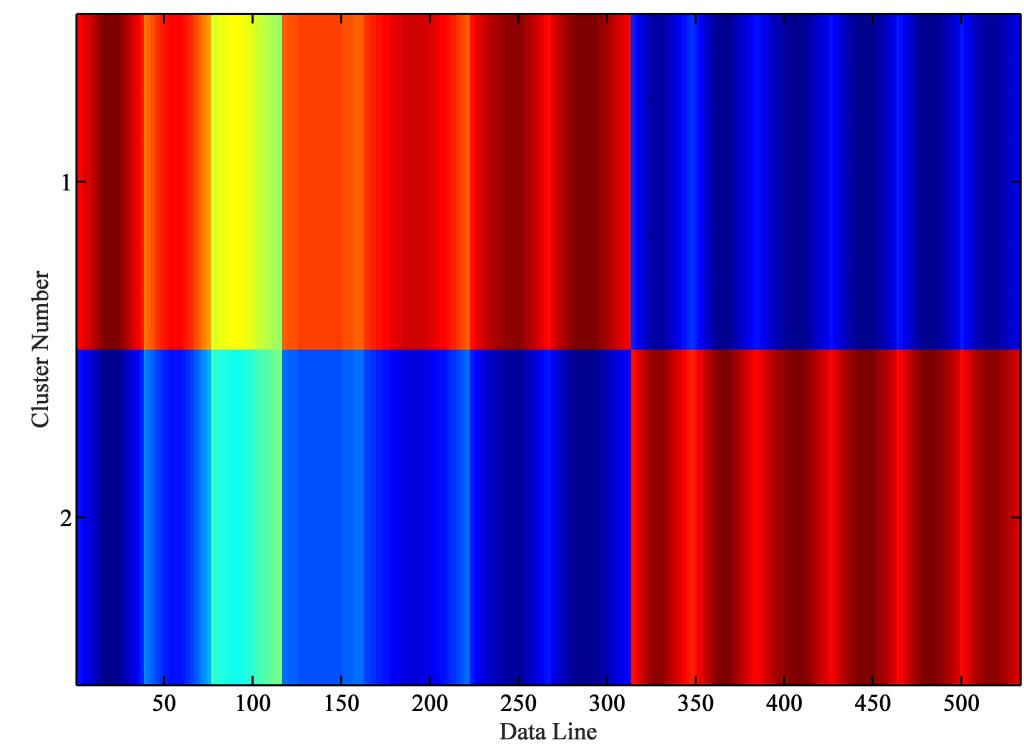

Supplement: Supplemental Material [file TBBE_A_1621206_SM3139.zip › Supplementary Figure 15.jpg]

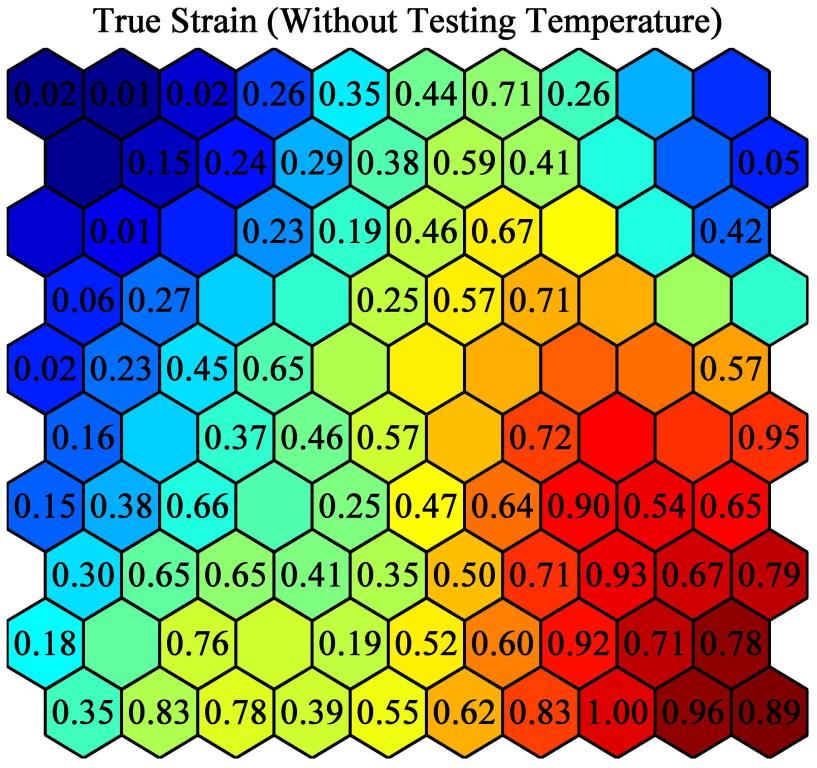

Supplement: Supplemental Material [file TBBE_A_1621206_SM3139.zip › Supplementary Figure 2(a).jpg]

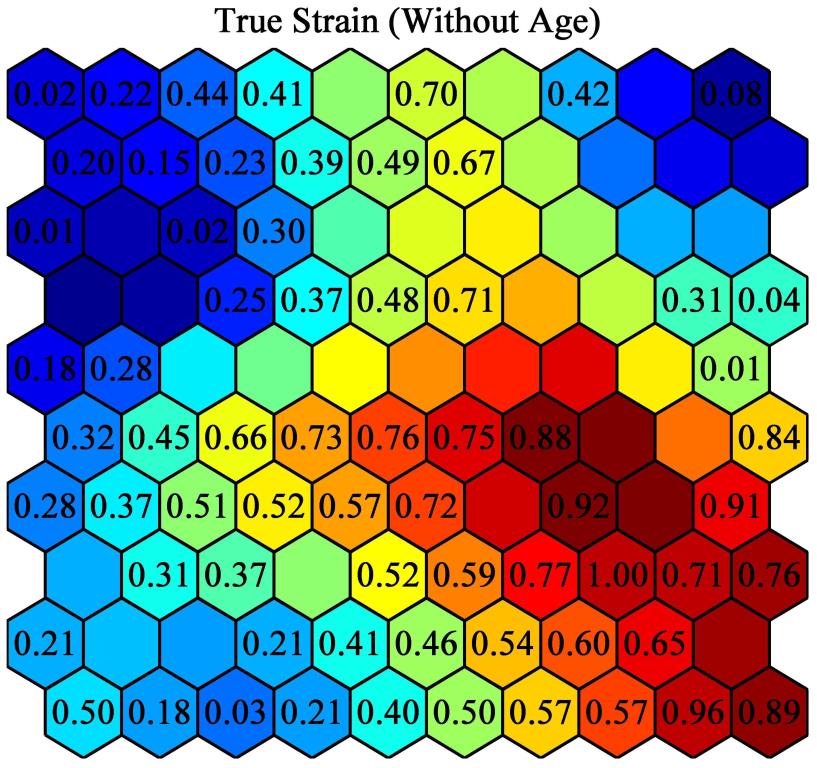

Supplement: Supplemental Material [file TBBE_A_1621206_SM3139.zip › Supplementary Figure 2(b).jpg]

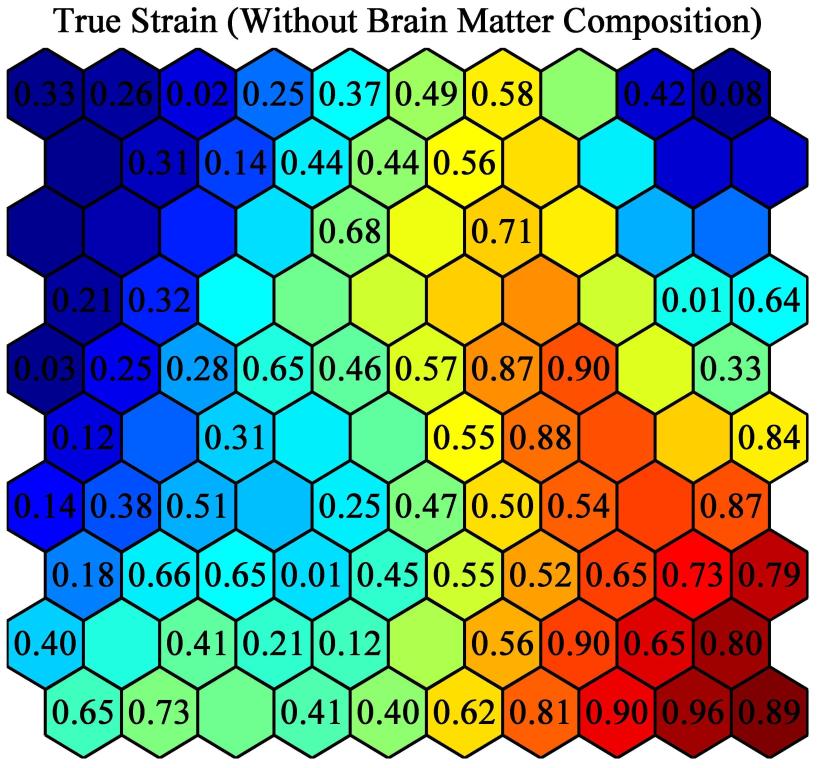

Supplement: Supplemental Material [file TBBE_A_1621206_SM3139.zip › Supplementary Figure 2(c).jpg]

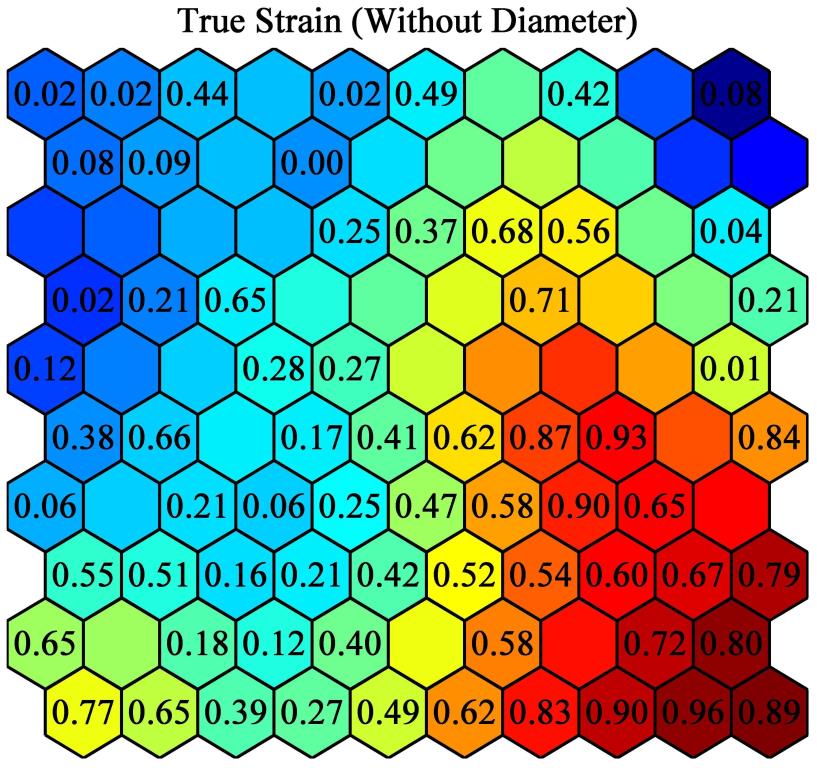

Supplement: Supplemental Material [file TBBE_A_1621206_SM3139.zip › Supplementary Figure 2(d).jpg]

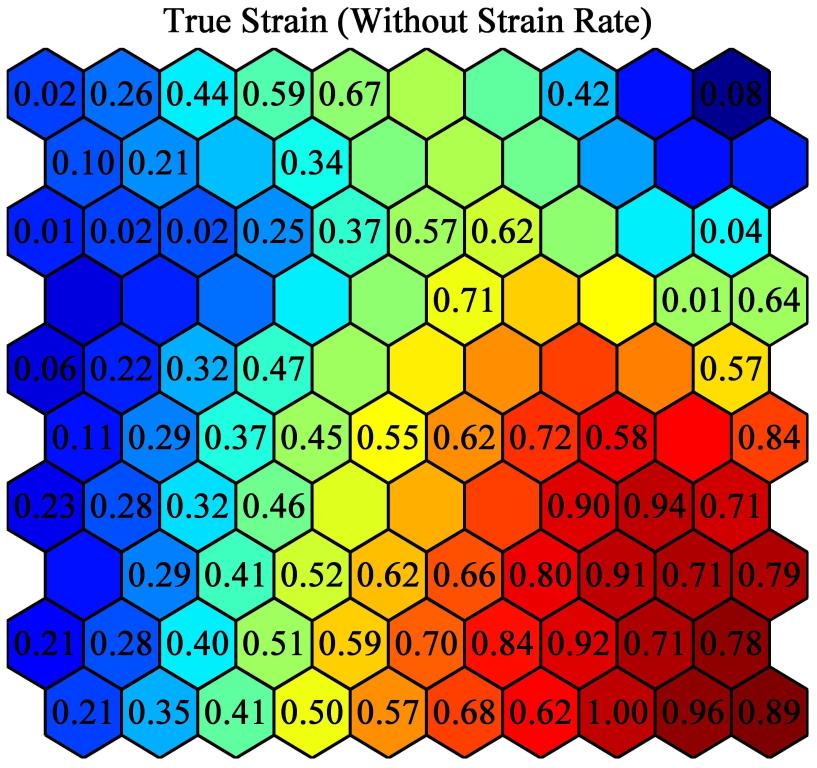

Supplement: Supplemental Material [file TBBE_A_1621206_SM3139.zip › Supplementary Figure 2(e).jpg]

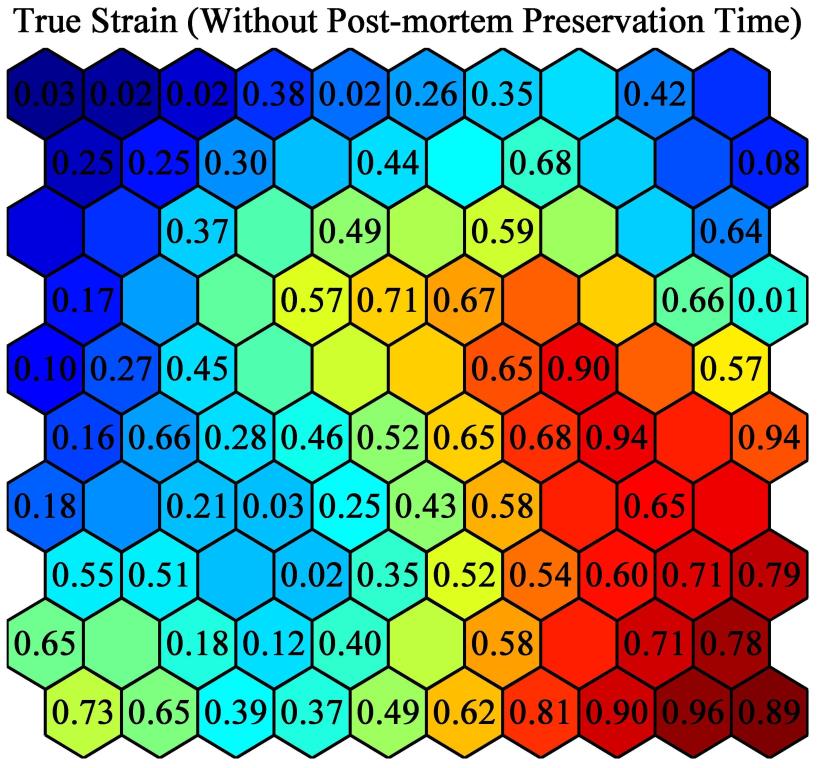

Supplement: Supplemental Material [file TBBE_A_1621206_SM3139.zip › Supplementary Figure 2(f).jpg]

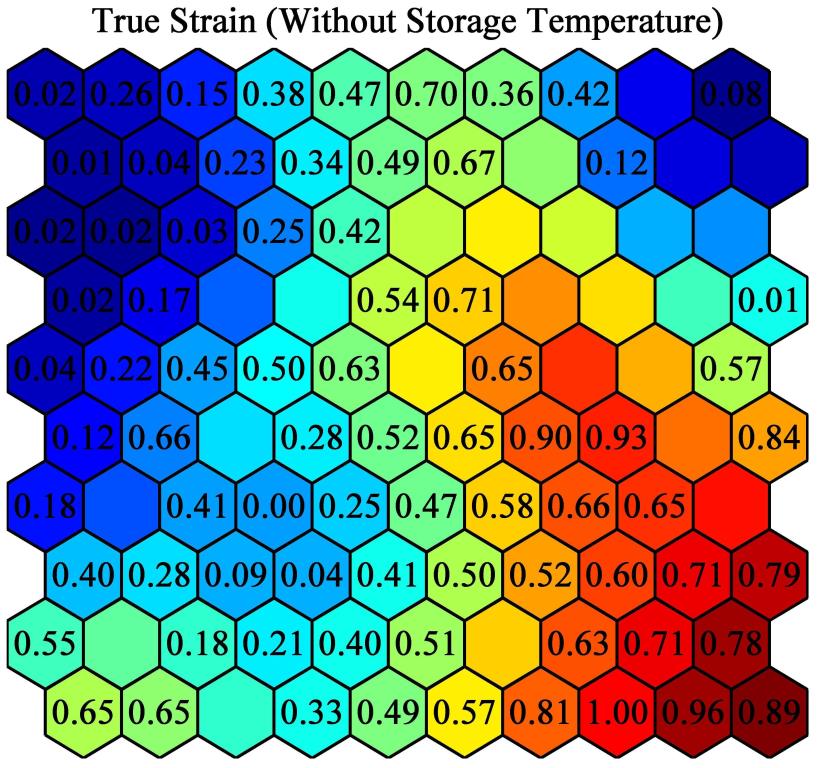

Supplement: Supplemental Material [file TBBE_A_1621206_SM3139.zip › Supplementary Figure 2(g).jpg]

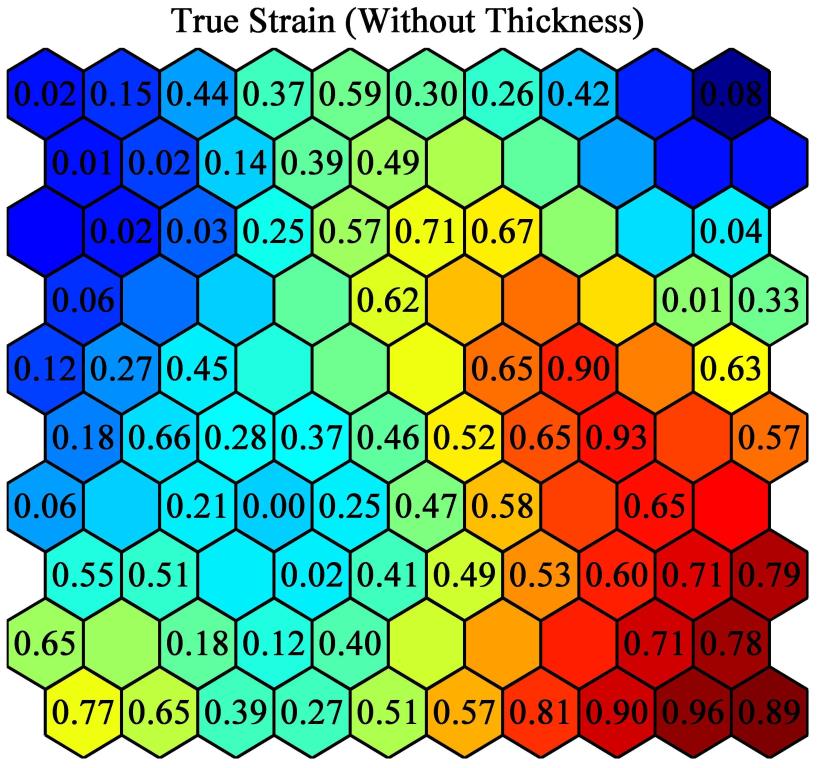

Supplement: Supplemental Material [file TBBE_A_1621206_SM3139.zip › Supplementary Figure 2(h).jpg]

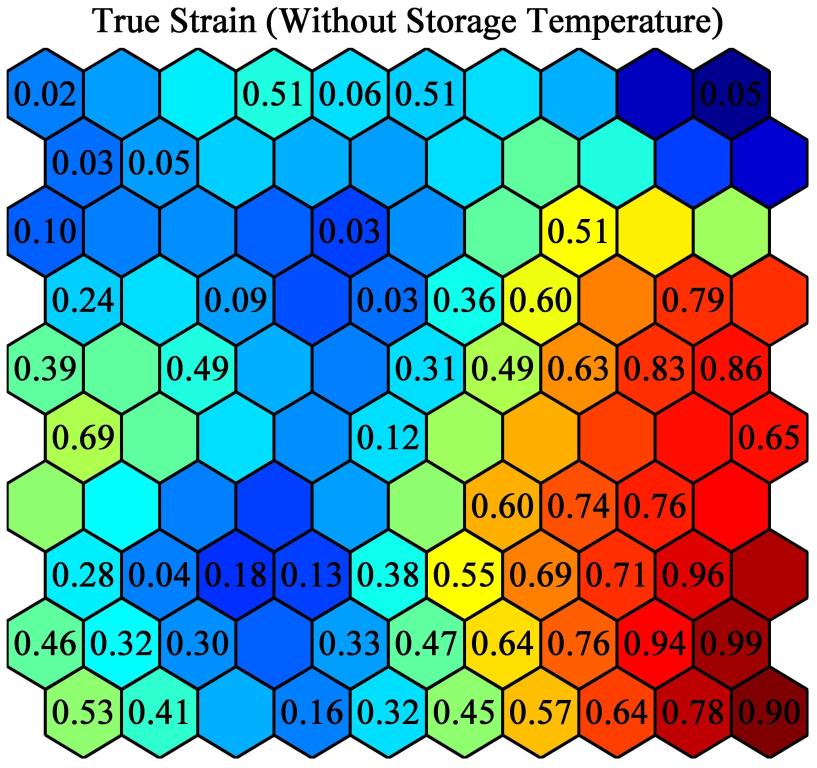

Supplement: Supplemental Material [file TBBE_A_1621206_SM3139.zip › Supplementary Figure 3(a).jpg]

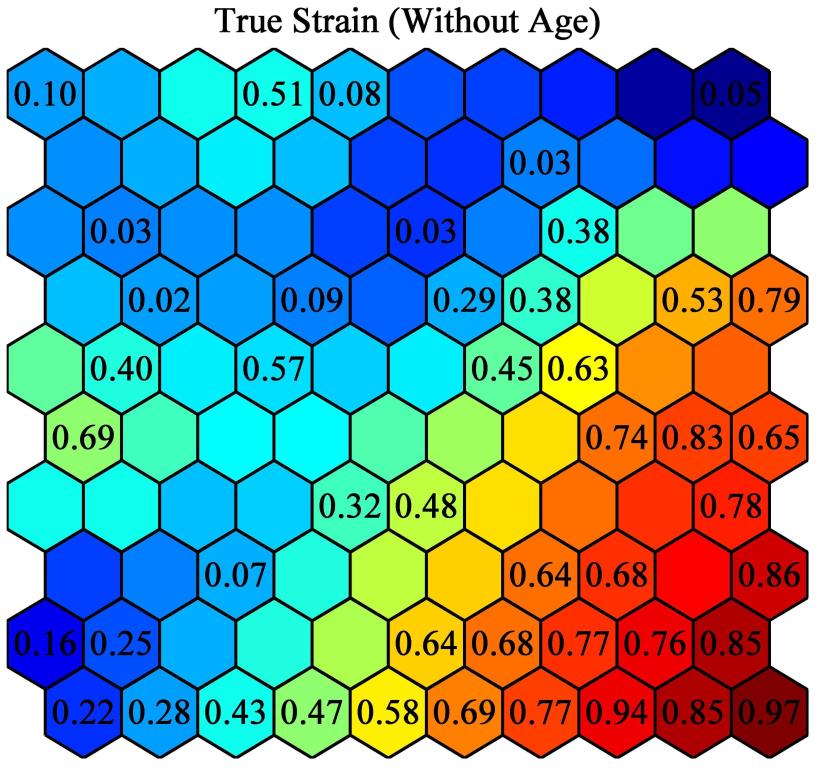

Supplement: Supplemental Material [file TBBE_A_1621206_SM3139.zip › Supplementary Figure 3(b).jpg]

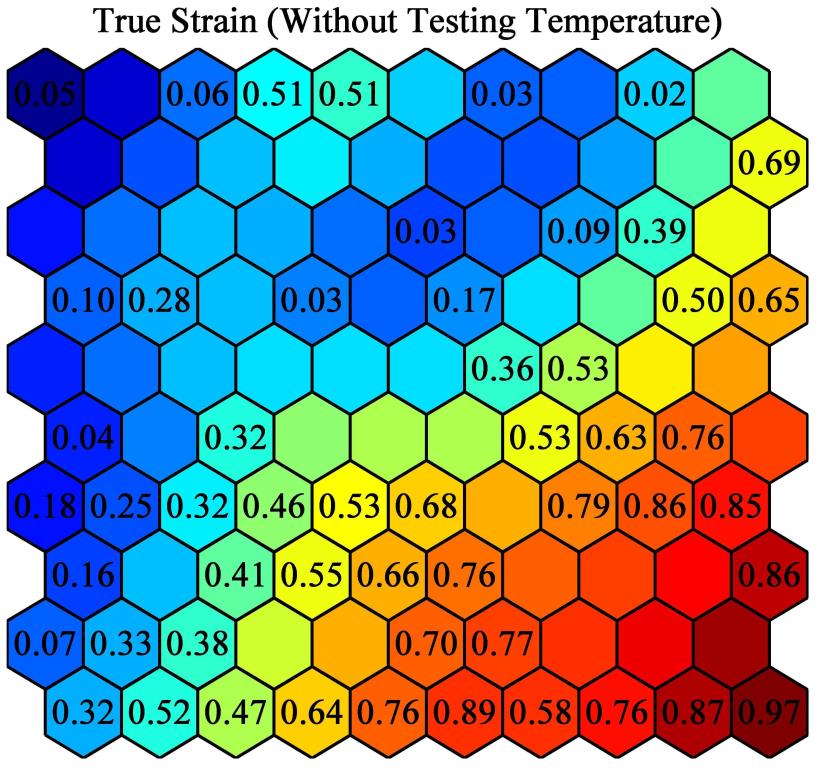

Supplement: Supplemental Material [file TBBE_A_1621206_SM3139.zip › Supplementary Figure 3(c).jpg]

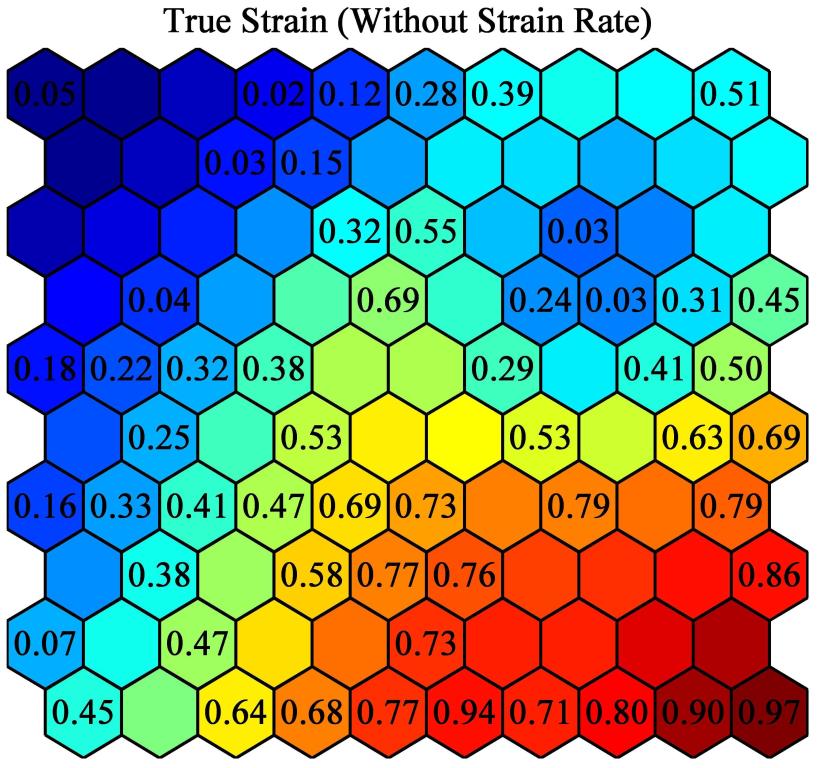

Supplement: Supplemental Material [file TBBE_A_1621206_SM3139.zip › Supplementary Figure 3(d).jpg]

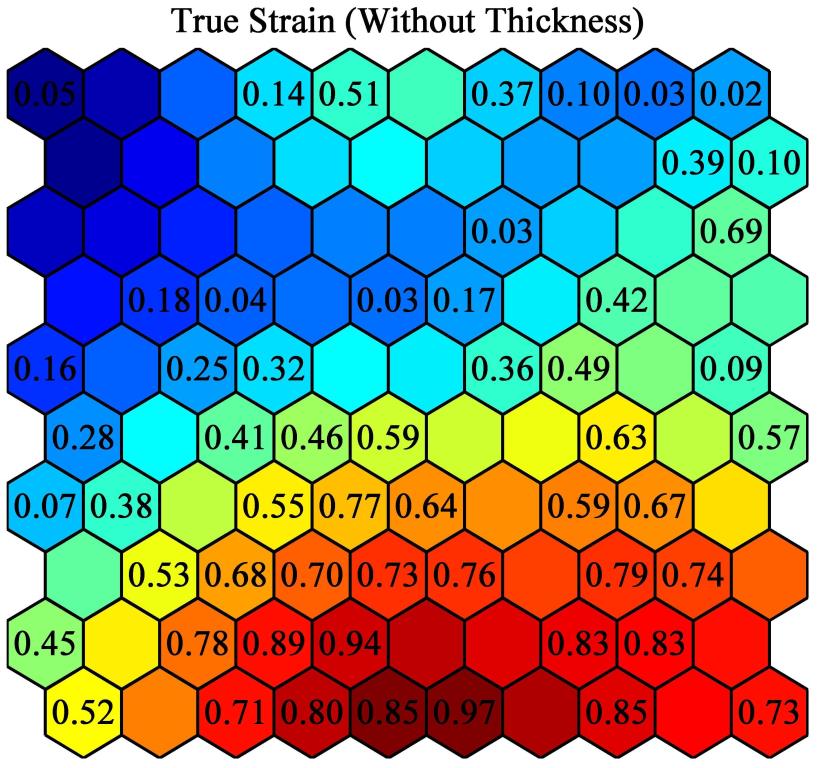

Supplement: Supplemental Material [file TBBE_A_1621206_SM3139.zip › Supplementary Figure 3(e).jpg]

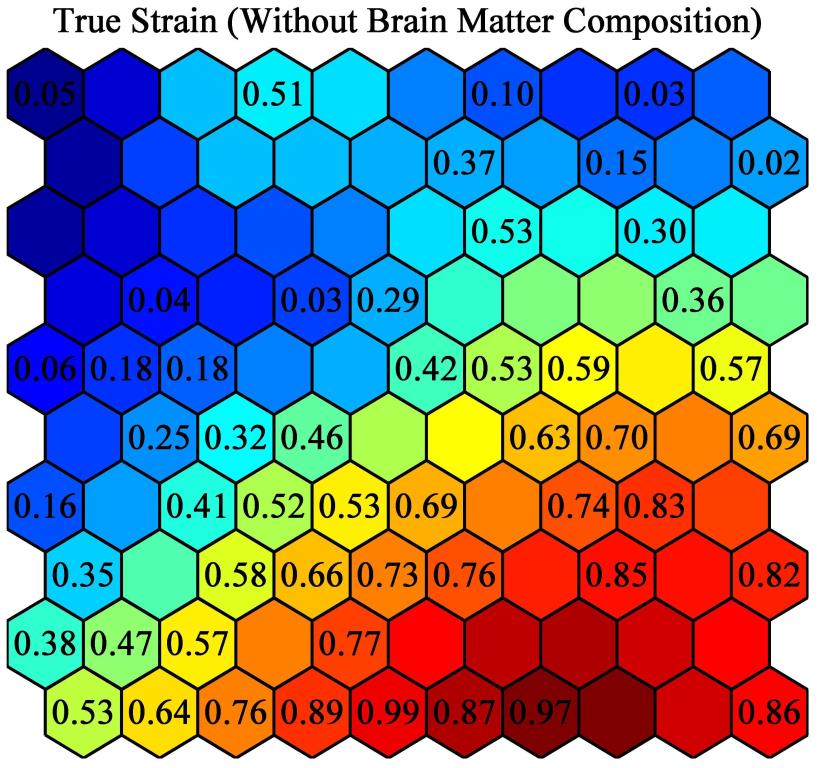

Supplement: Supplemental Material [file TBBE_A_1621206_SM3139.zip › Supplementary Figure 3(f).jpg]

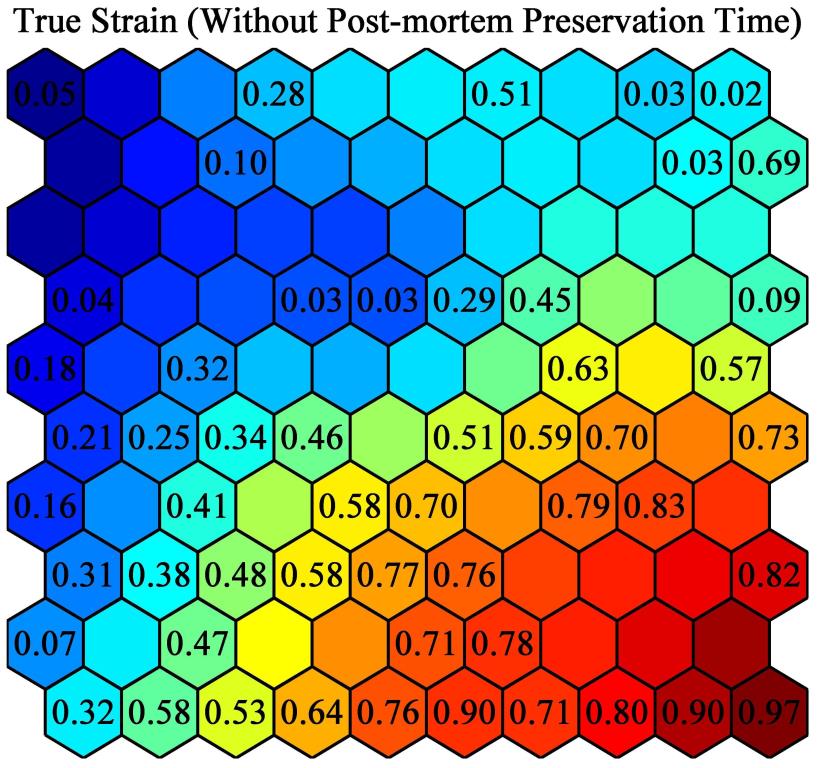

Supplement: Supplemental Material [file TBBE_A_1621206_SM3139.zip › Supplementary Figure 3(g).jpg]

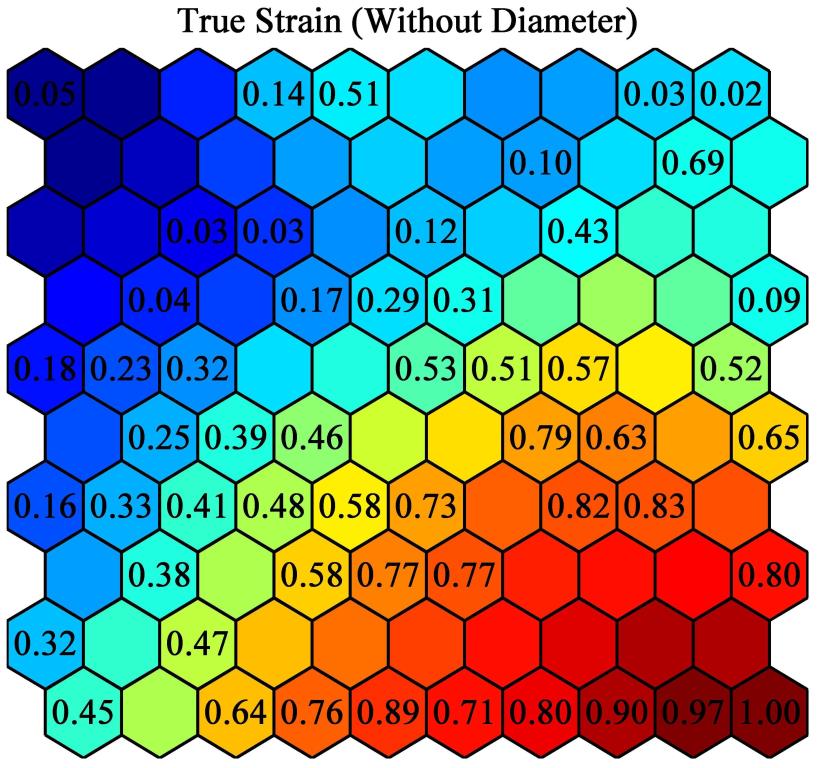

Supplement: Supplemental Material [file TBBE_A_1621206_SM3139.zip › Supplementary Figure 3(h).jpg]

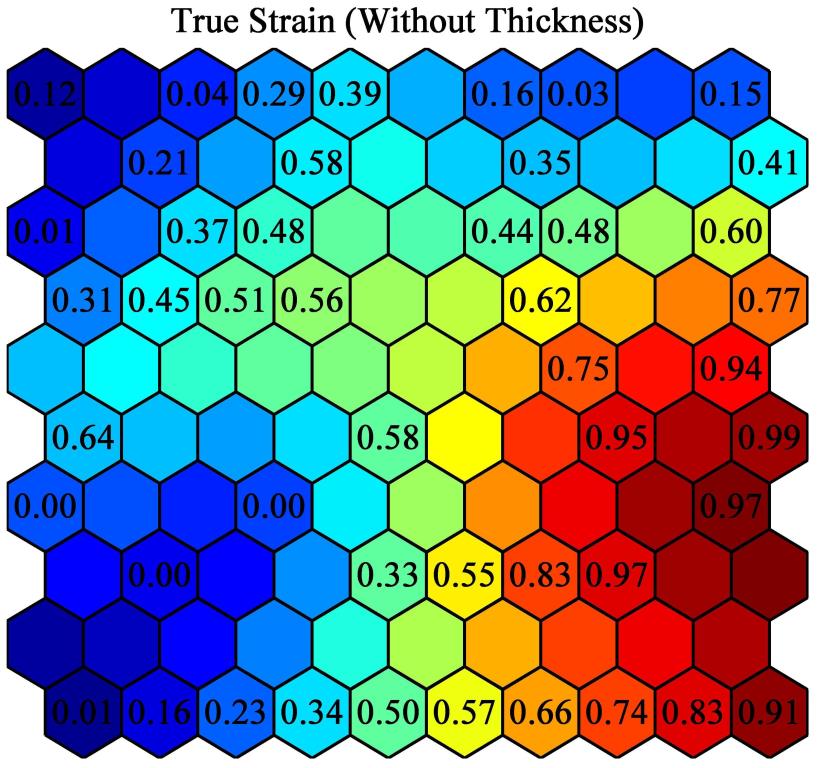

Supplement: Supplemental Material [file TBBE_A_1621206_SM3139.zip › Supplementary Figure 4(a).jpg]

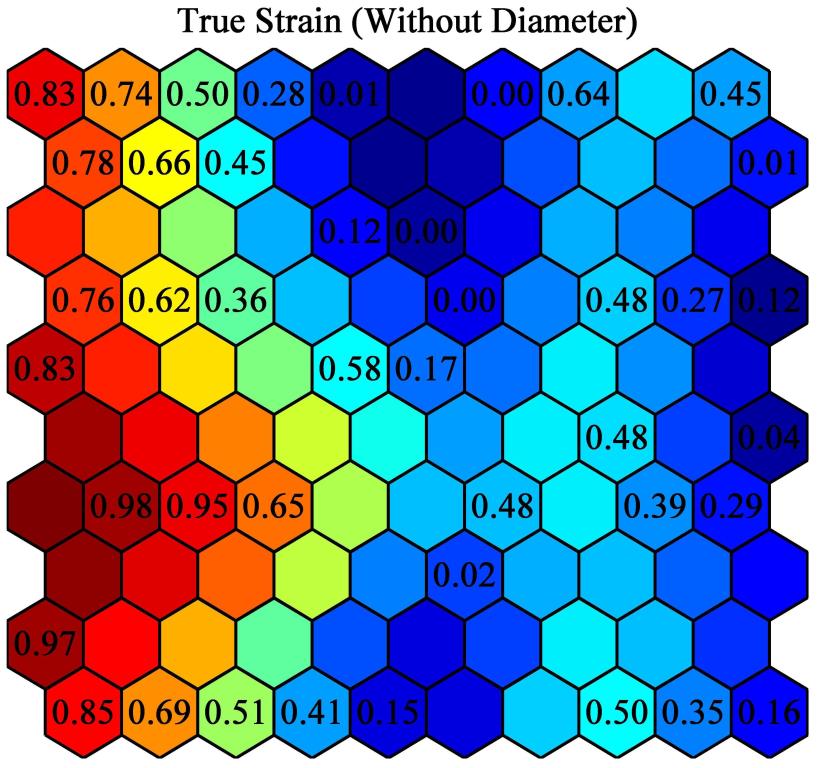

Supplement: Supplemental Material [file TBBE_A_1621206_SM3139.zip › Supplementary Figure 4(b).jpg]

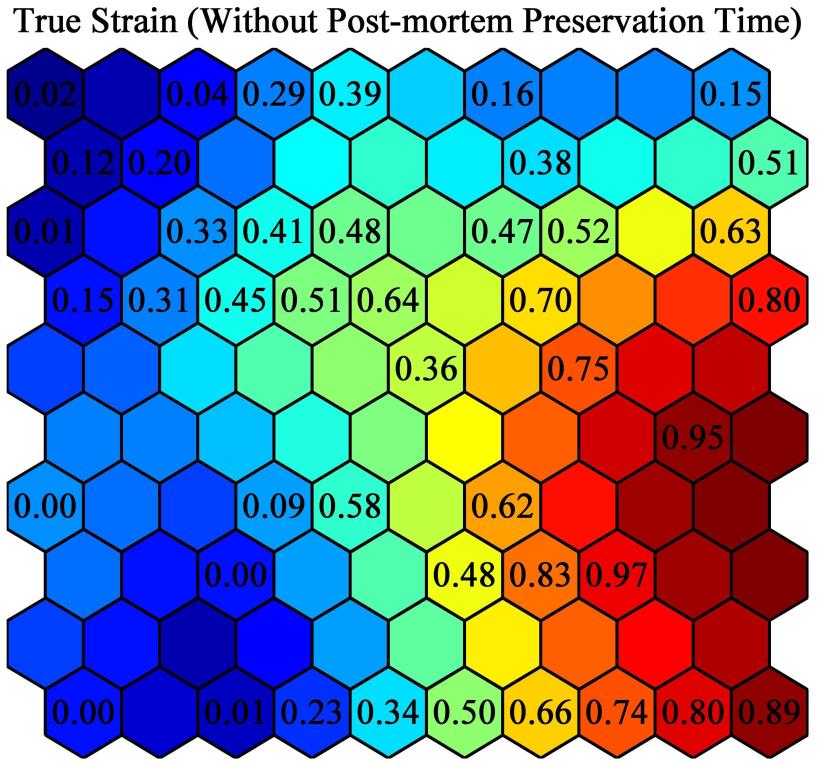

Supplement: Supplemental Material [file TBBE_A_1621206_SM3139.zip › Supplementary Figure 4(c).jpg]

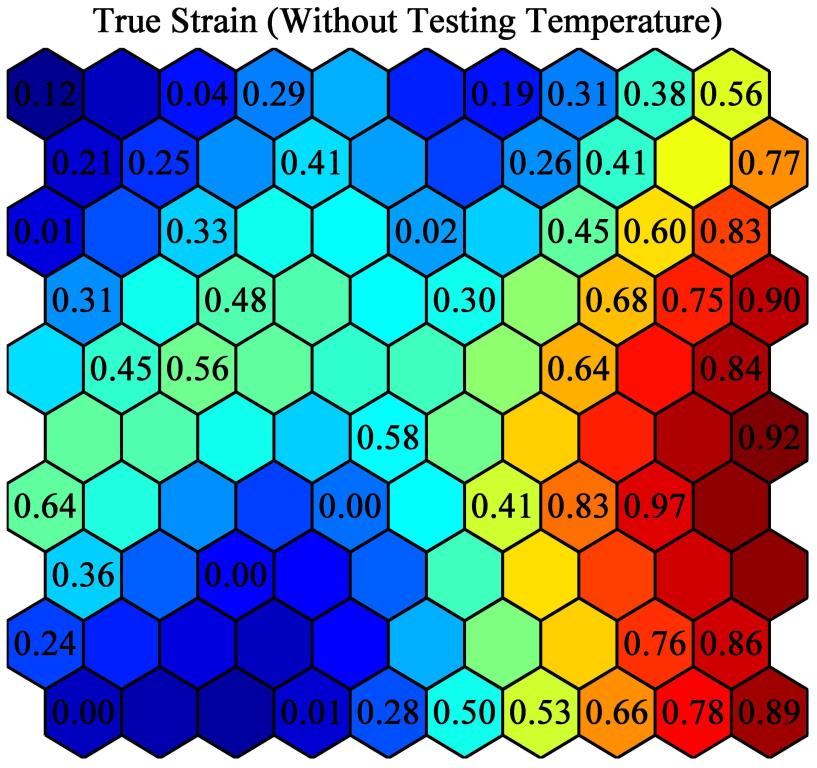

Supplement: Supplemental Material [file TBBE_A_1621206_SM3139.zip › Supplementary Figure 4(d).jpg]

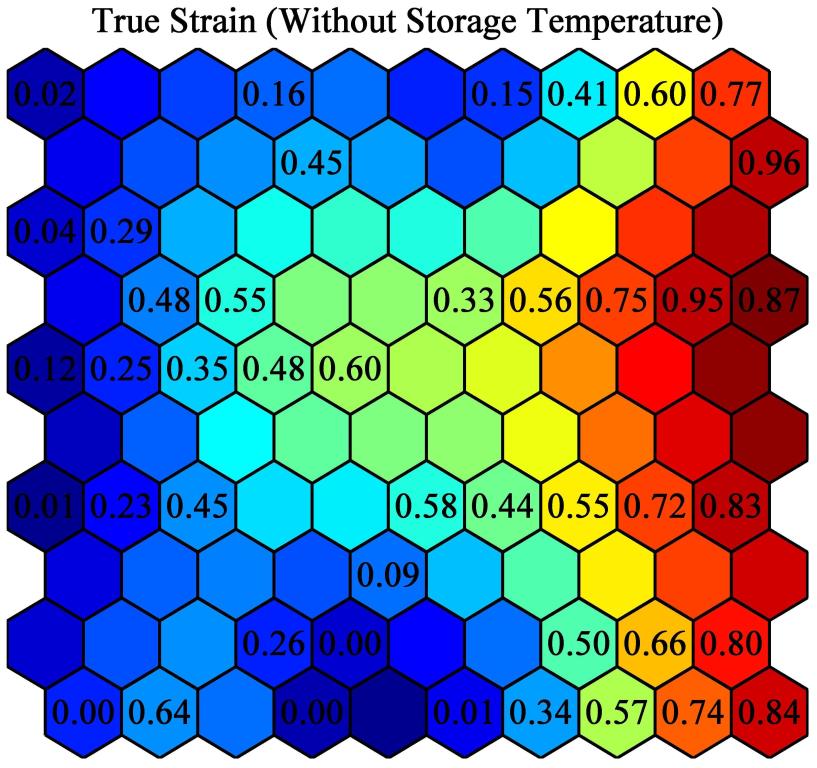

Supplement: Supplemental Material [file TBBE_A_1621206_SM3139.zip › Supplementary Figure 4(e).jpg]

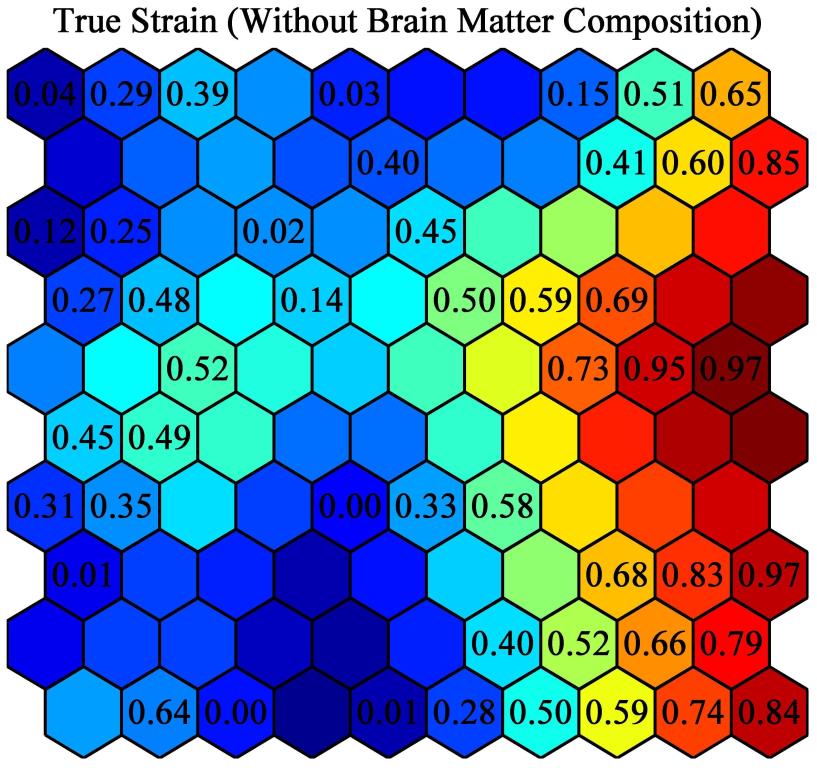

Supplement: Supplemental Material [file TBBE_A_1621206_SM3139.zip › Supplementary Figure 4(f).jpg]

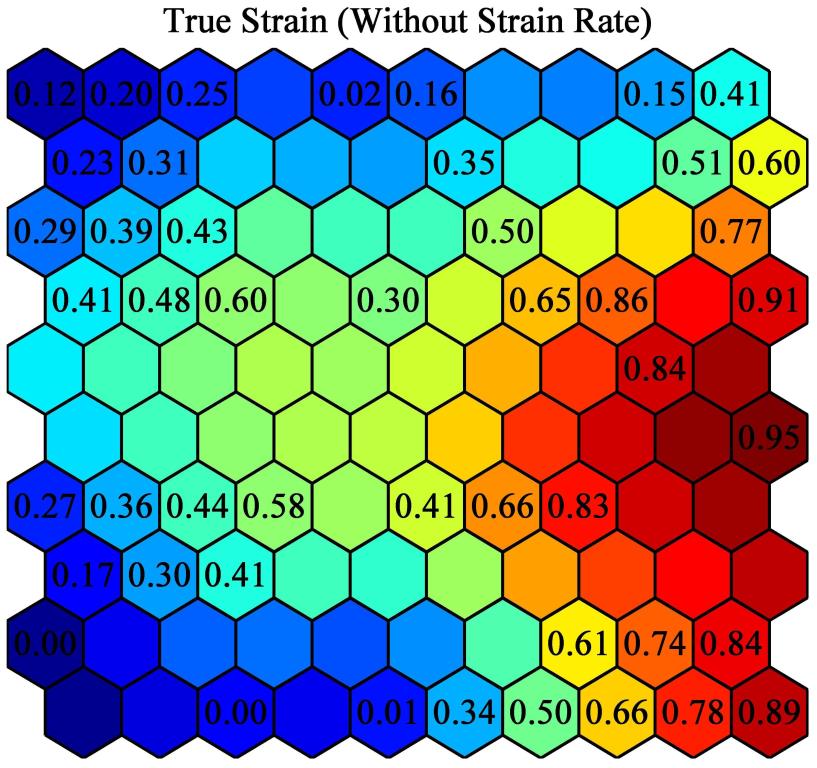

Supplement: Supplemental Material [file TBBE_A_1621206_SM3139.zip › Supplementary Figure 4(g).jpg]

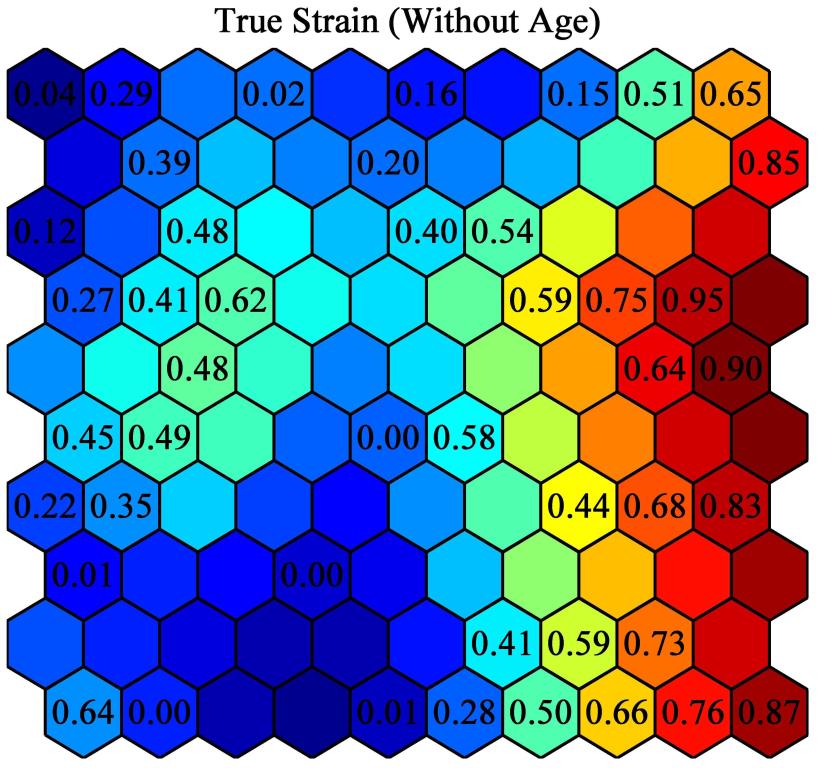

Supplement: Supplemental Material [file TBBE_A_1621206_SM3139.zip › Supplementary Figure 4(h).jpg]

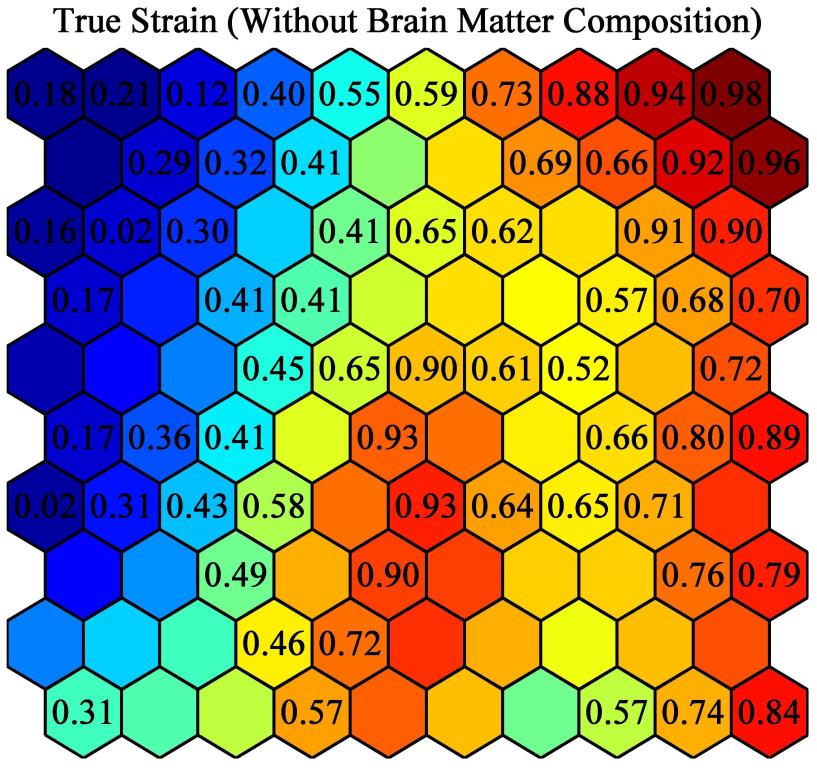

Supplement: Supplemental Material [file TBBE_A_1621206_SM3139.zip › Supplementary Figure 5(a).jpg]

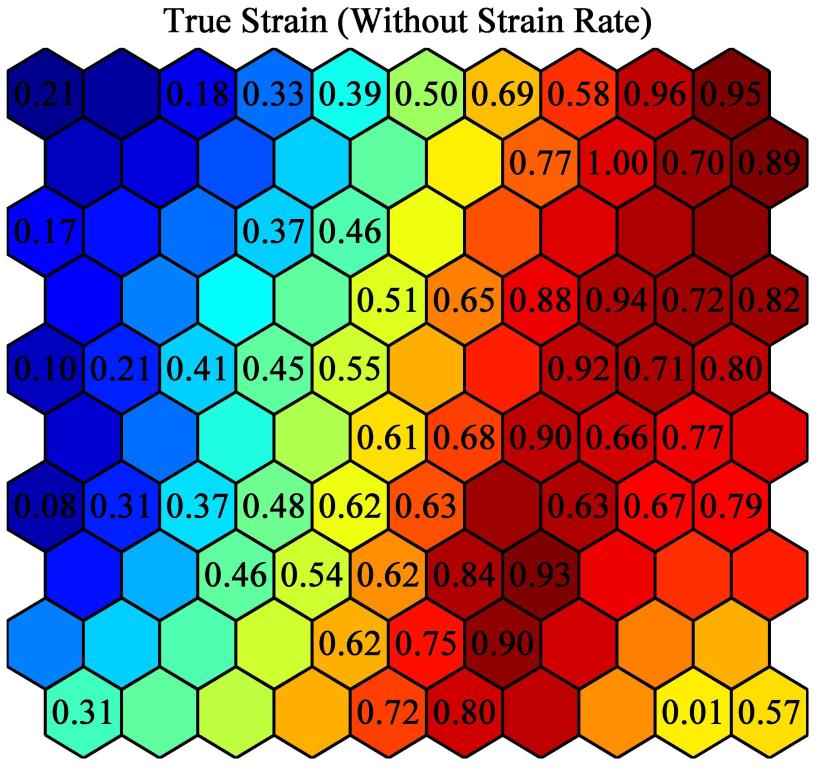

Supplement: Supplemental Material [file TBBE_A_1621206_SM3139.zip › Supplementary Figure 5(b).jpg]

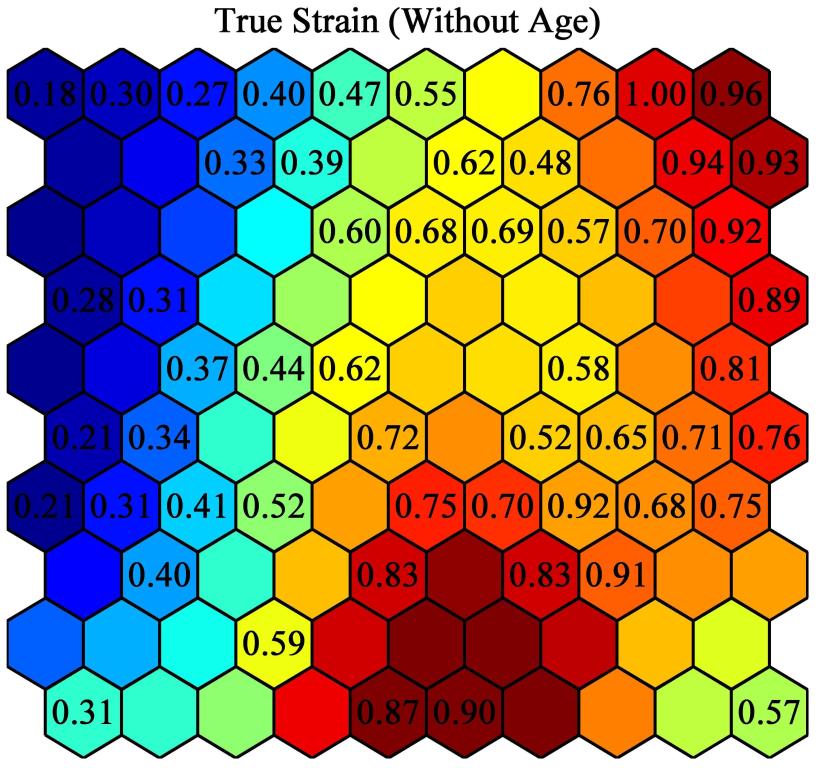

Supplement: Supplemental Material [file TBBE_A_1621206_SM3139.zip › Supplementary Figure 5(c).jpg]

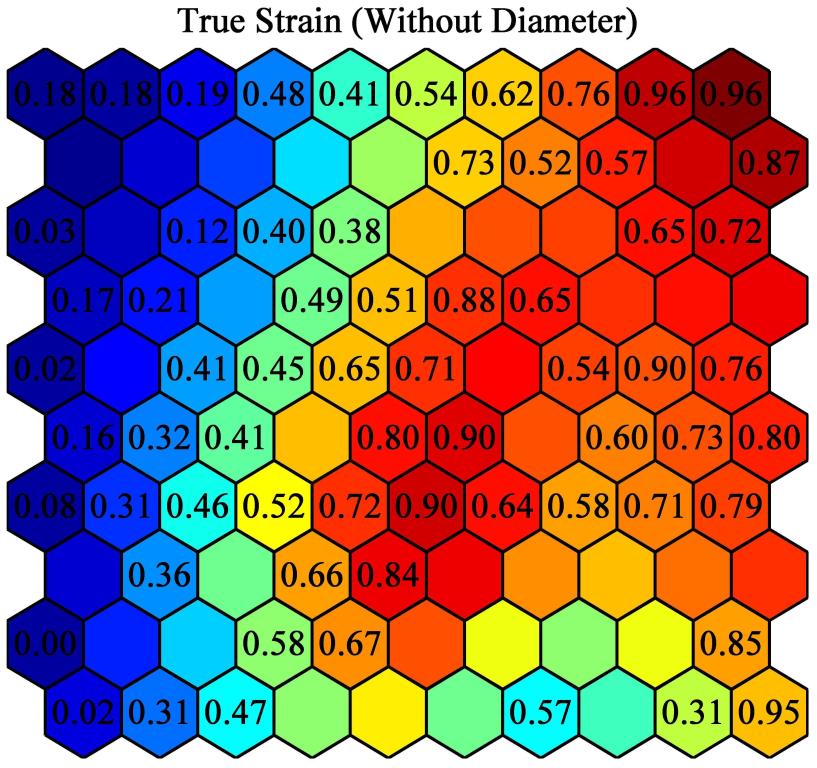

Supplement: Supplemental Material [file TBBE_A_1621206_SM3139.zip › Supplementary Figure 5(d).jpg]

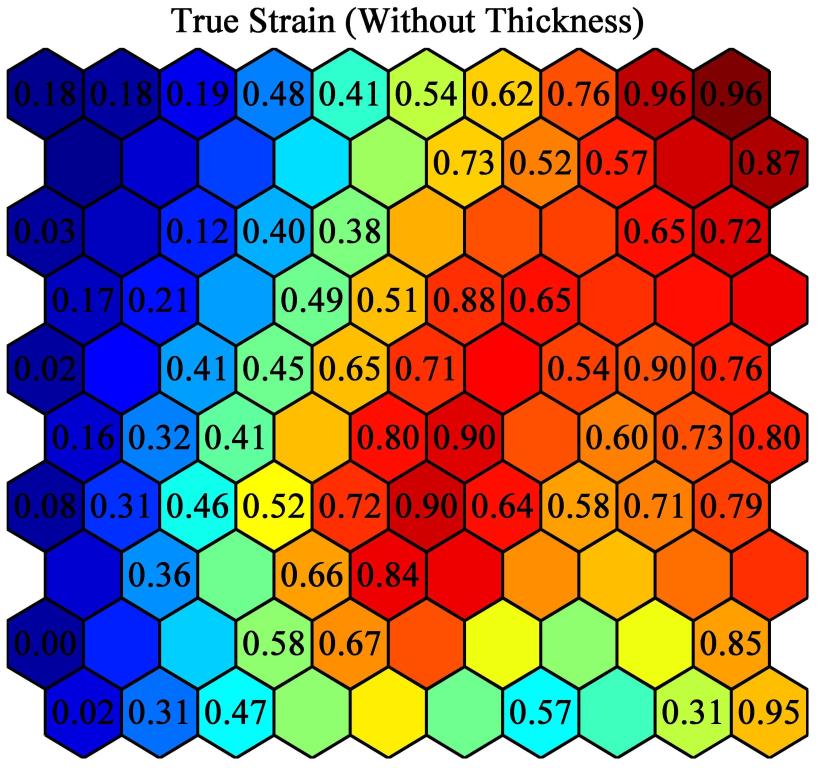

Supplement: Supplemental Material [file TBBE_A_1621206_SM3139.zip › Supplementary Figure 5(e).jpg]

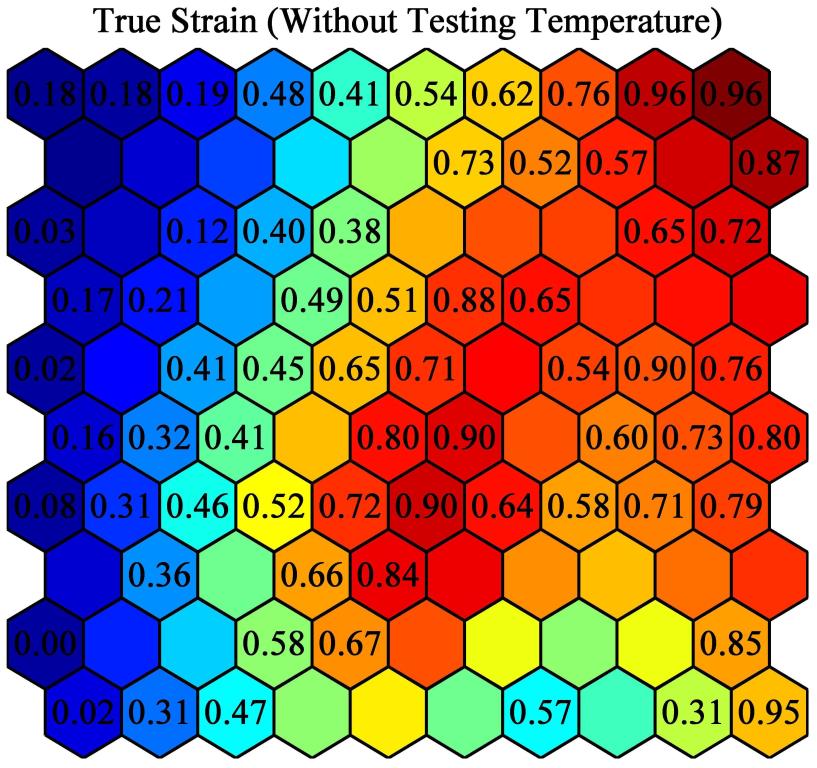

Supplement: Supplemental Material [file TBBE_A_1621206_SM3139.zip › Supplementary Figure 5(f).jpg]

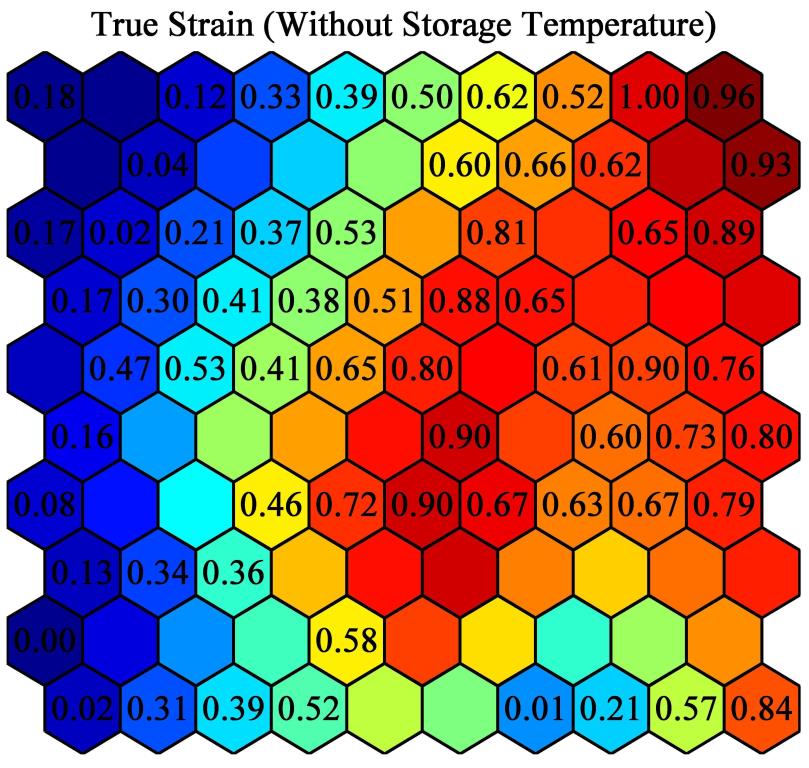

Supplement: Supplemental Material [file TBBE_A_1621206_SM3139.zip › Supplementary Figure 5(g).jpg]

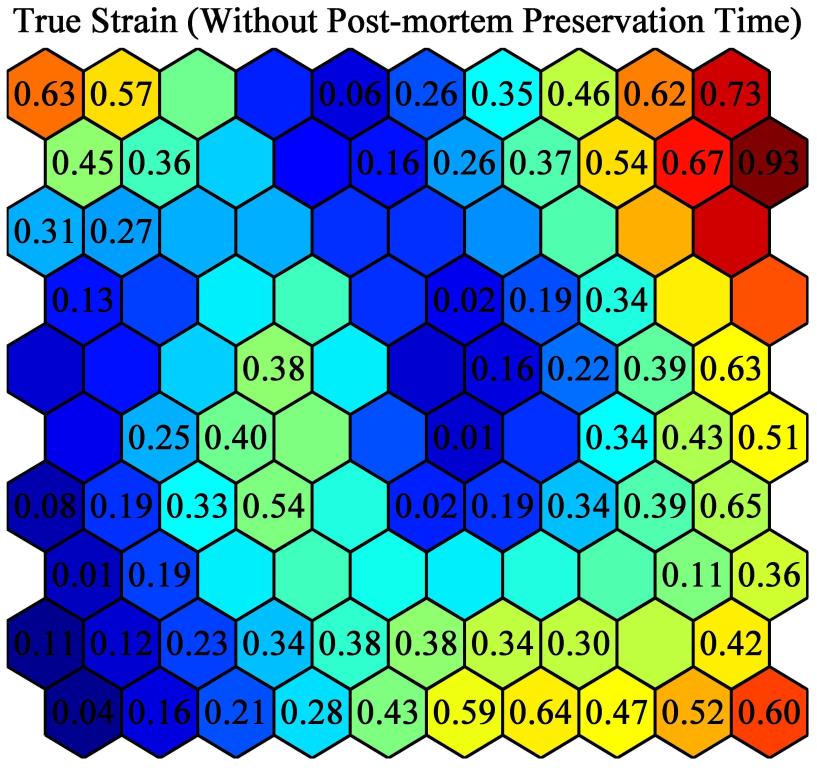

Supplement: Supplemental Material [file TBBE_A_1621206_SM3139.zip › Supplementary Figure 6(a).jpg]

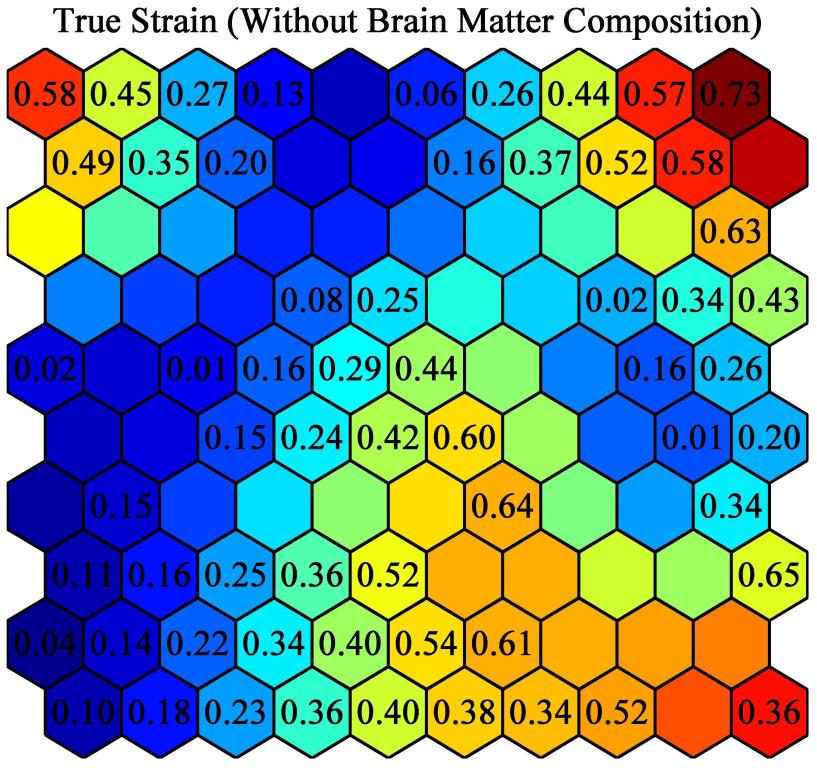

Supplement: Supplemental Material [file TBBE_A_1621206_SM3139.zip › Supplementary Figure 6(b).jpg]

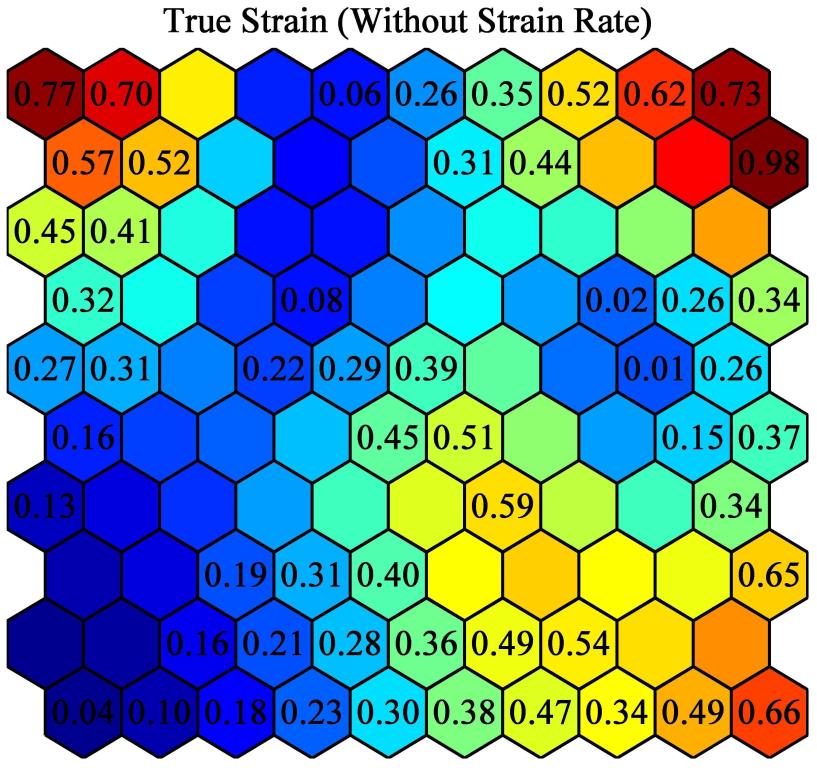

Supplement: Supplemental Material [file TBBE_A_1621206_SM3139.zip › Supplementary Figure 6(c).jpg]

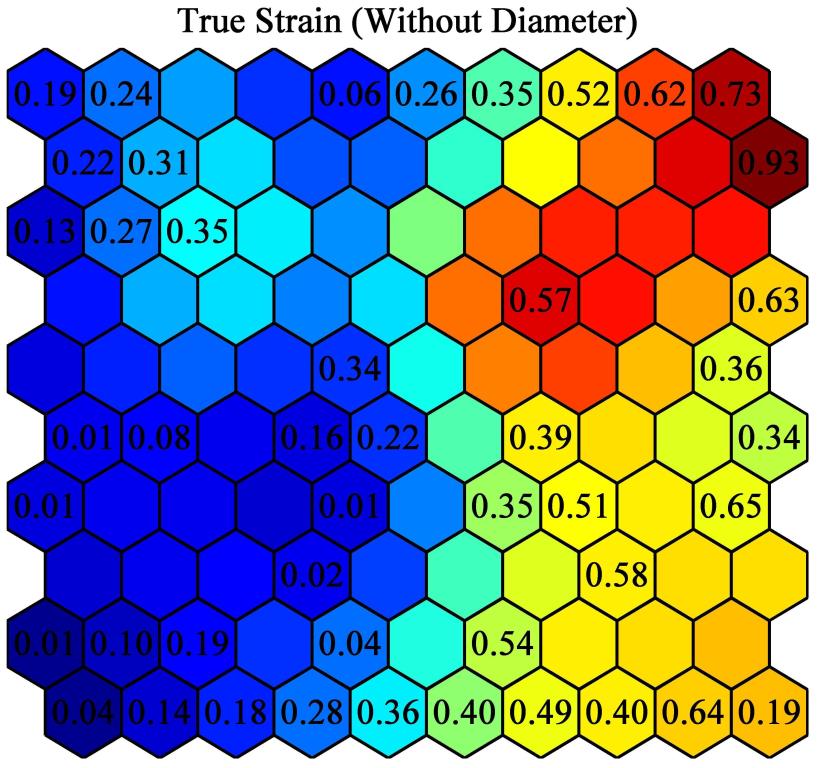

Supplement: Supplemental Material [file TBBE_A_1621206_SM3139.zip › Supplementary Figure 6(d).jpg]

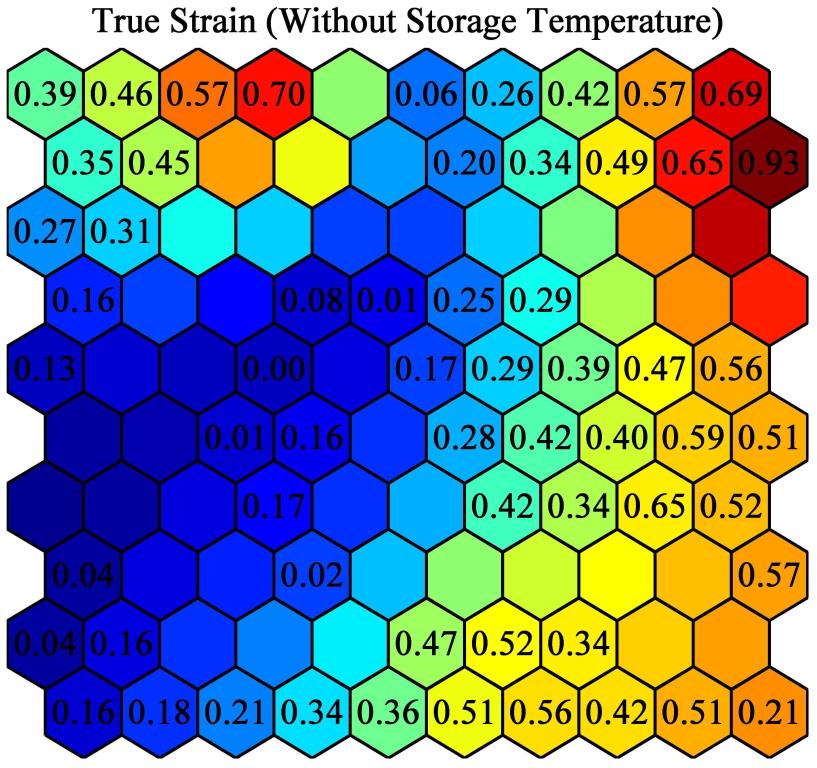

Supplement: Supplemental Material [file TBBE_A_1621206_SM3139.zip › Supplementary Figure 6(e).jpg]

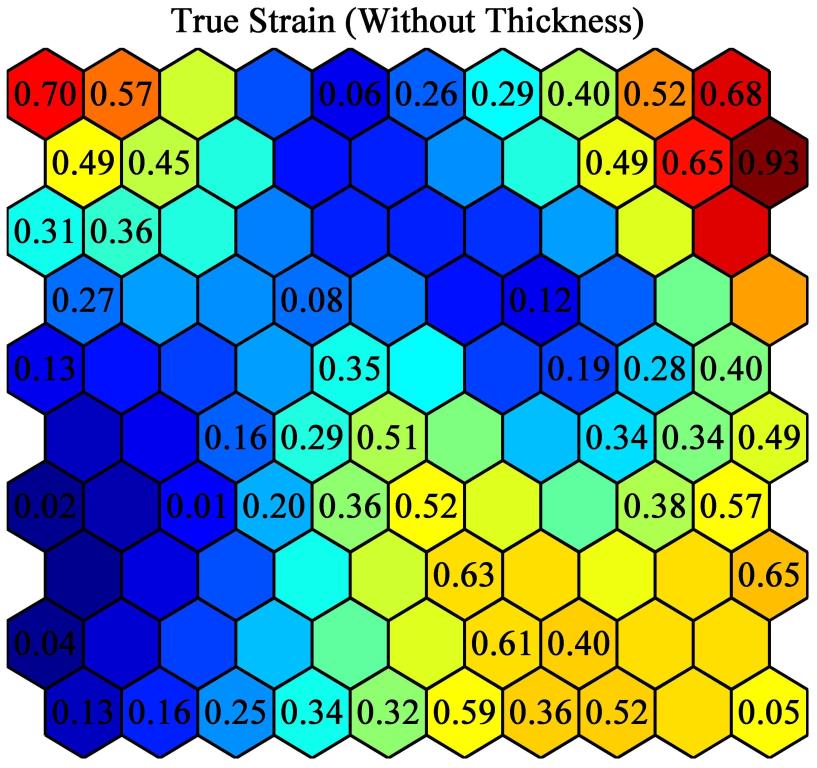

Supplement: Supplemental Material [file TBBE_A_1621206_SM3139.zip › Supplementary Figure 6(f).jpg]

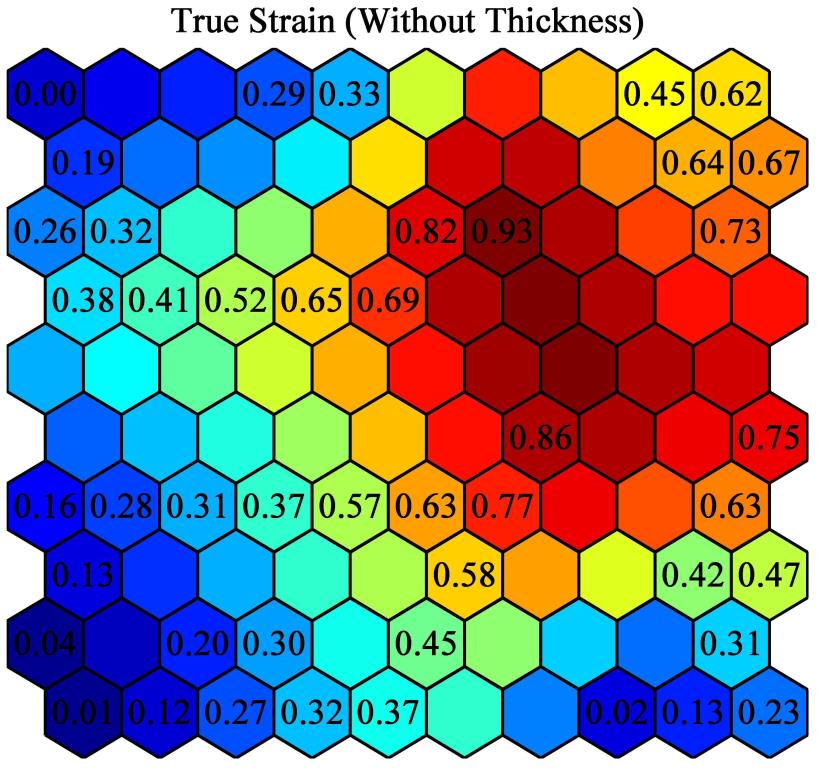

Supplement: Supplemental Material [file TBBE_A_1621206_SM3139.zip › Supplementary Figure 7(a).jpg]

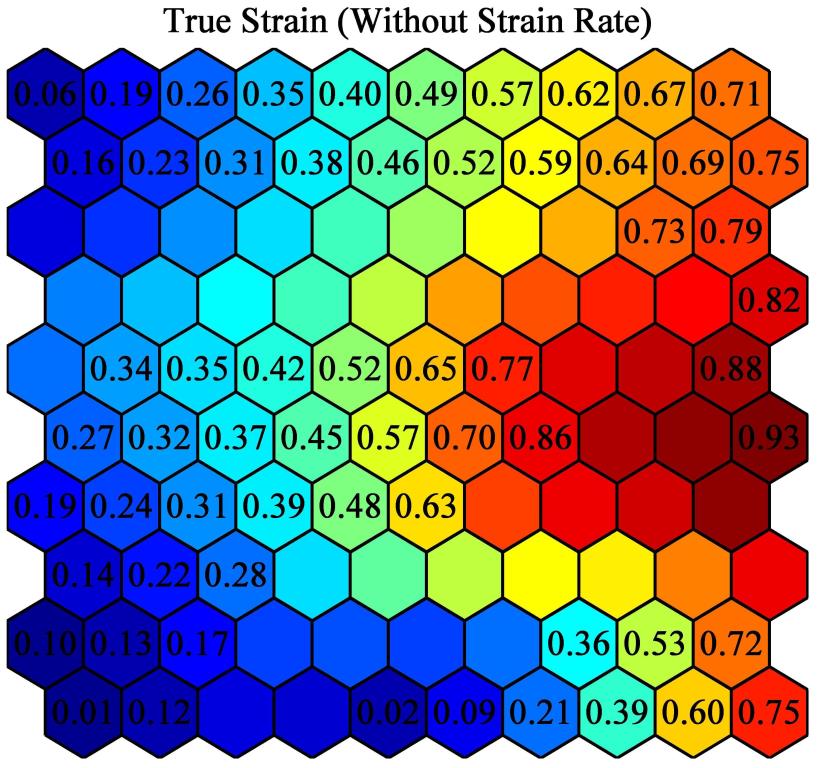

Supplement: Supplemental Material [file TBBE_A_1621206_SM3139.zip › Supplementary Figure 7(b).jpg]

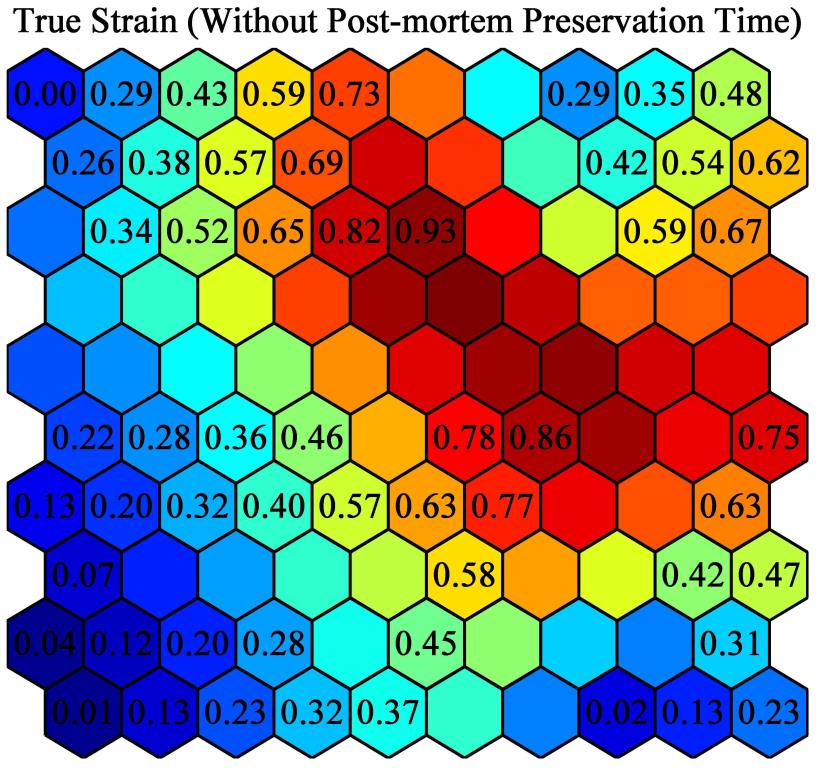

Supplement: Supplemental Material [file TBBE_A_1621206_SM3139.zip › Supplementary Figure 7(c).jpg]

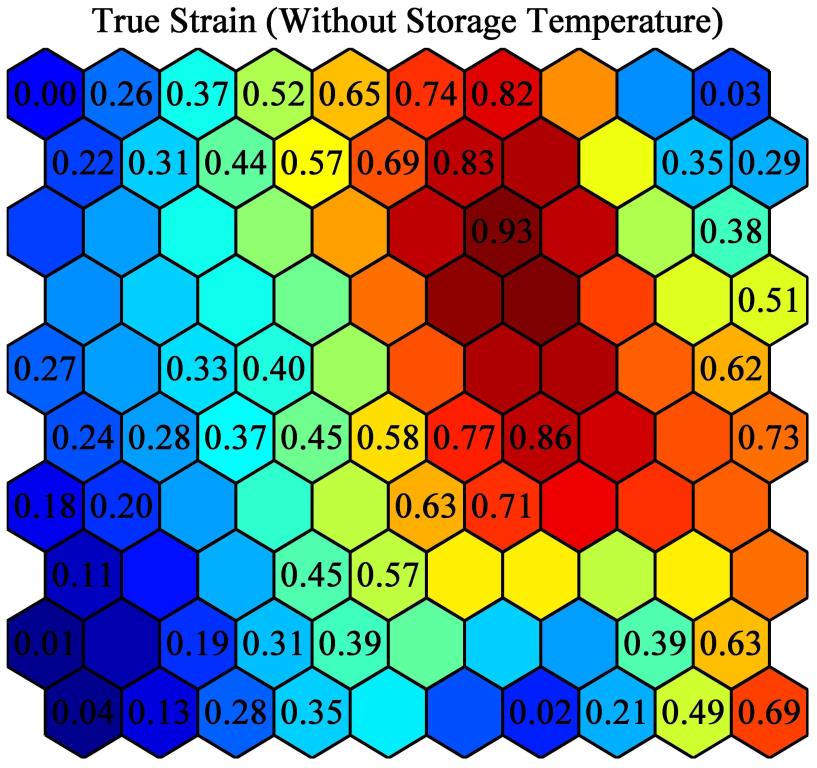

Supplement: Supplemental Material [file TBBE_A_1621206_SM3139.zip › Supplementary Figure 7(d).jpg]

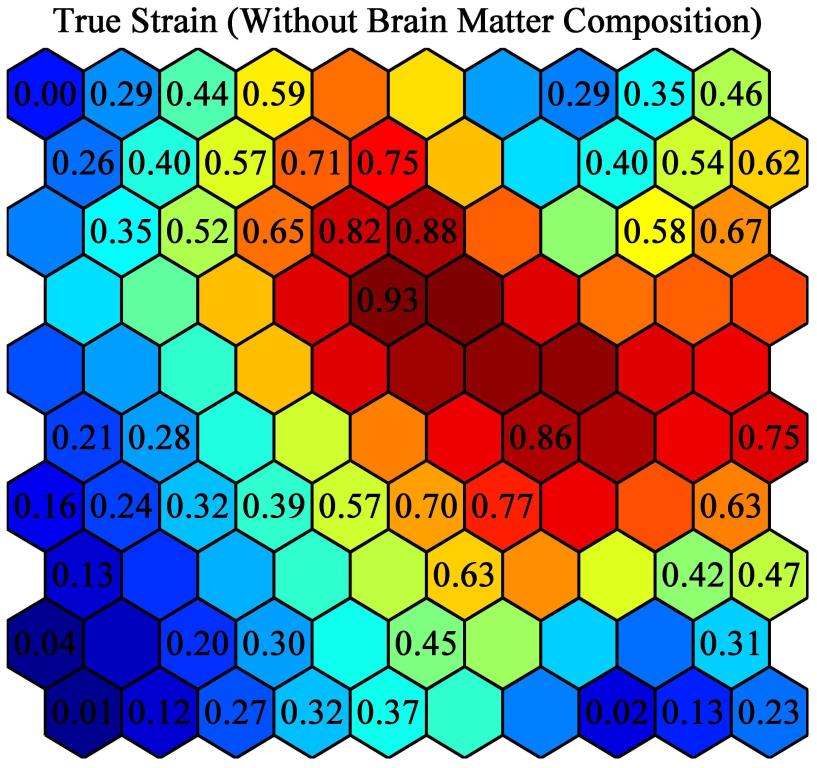

Supplement: Supplemental Material [file TBBE_A_1621206_SM3139.zip › Supplementary Figure 7(e).jpg]

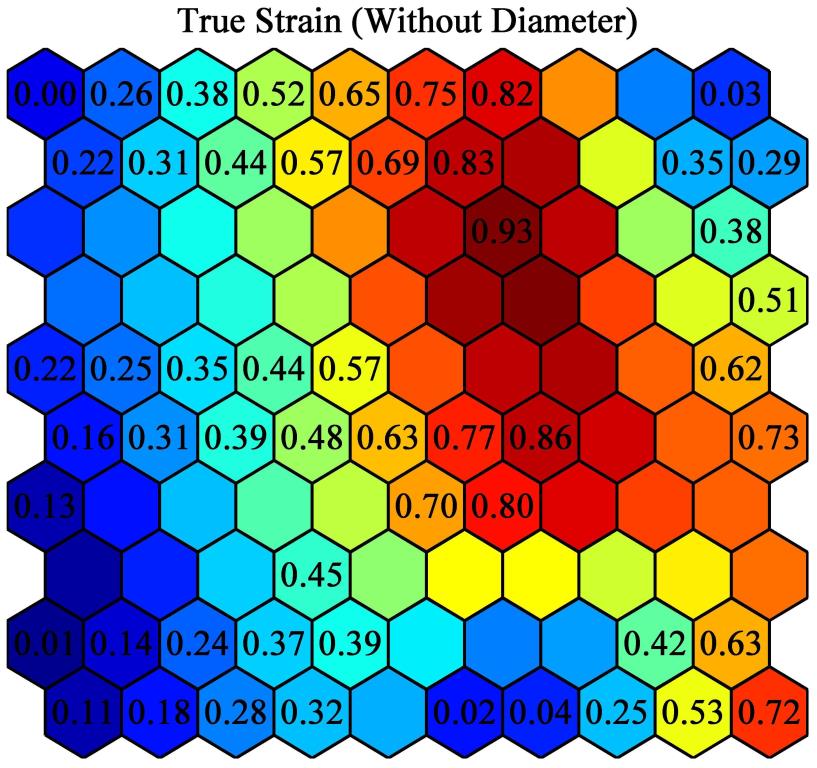

Supplement: Supplemental Material [file TBBE_A_1621206_SM3139.zip › Supplementary Figure 7(f).jpg]

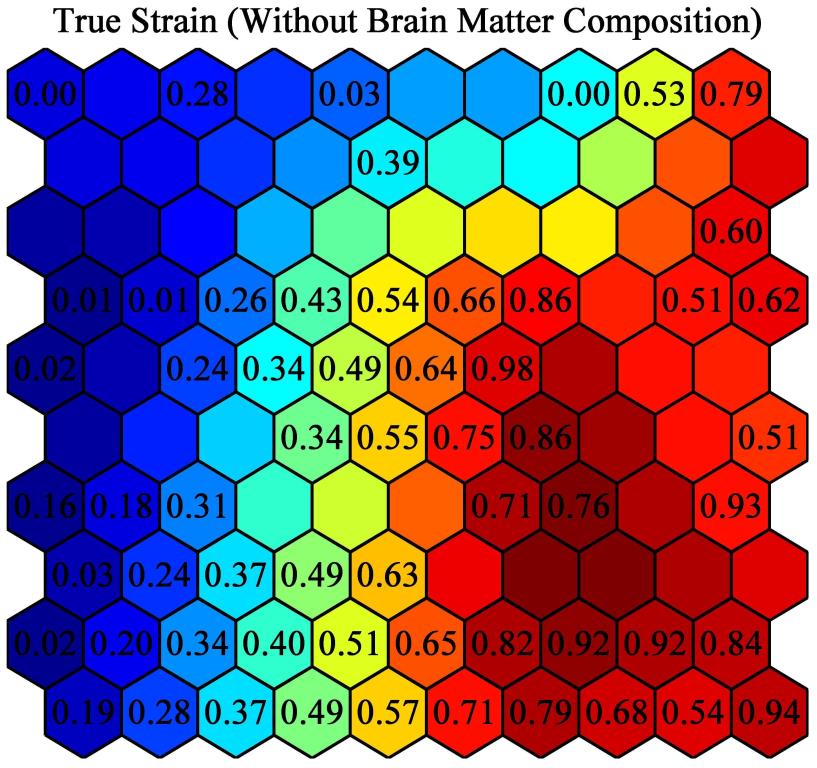

Supplement: Supplemental Material [file TBBE_A_1621206_SM3139.zip › Supplementary Figure 8(a).jpg]

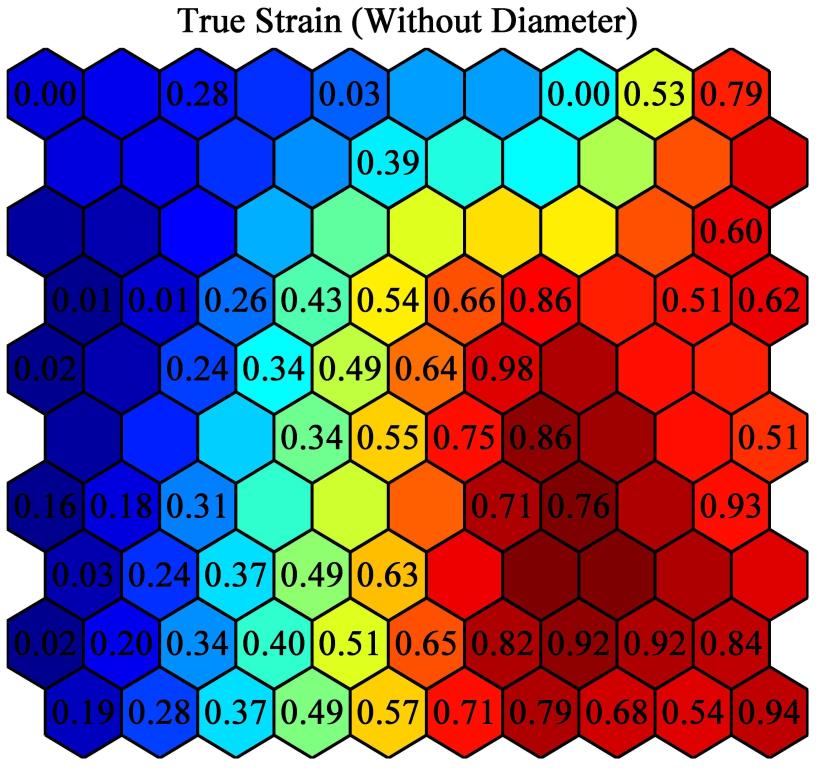

Supplement: Supplemental Material [file TBBE_A_1621206_SM3139.zip › Supplementary Figure 8(b).jpg]

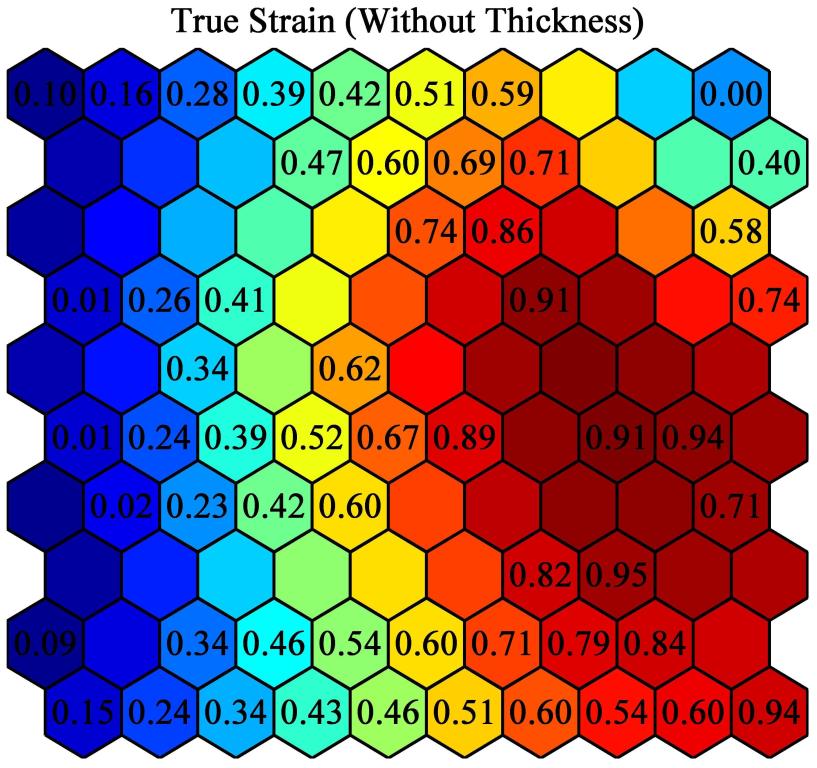

Supplement: Supplemental Material [file TBBE_A_1621206_SM3139.zip › Supplementary Figure 8(c).jpg]

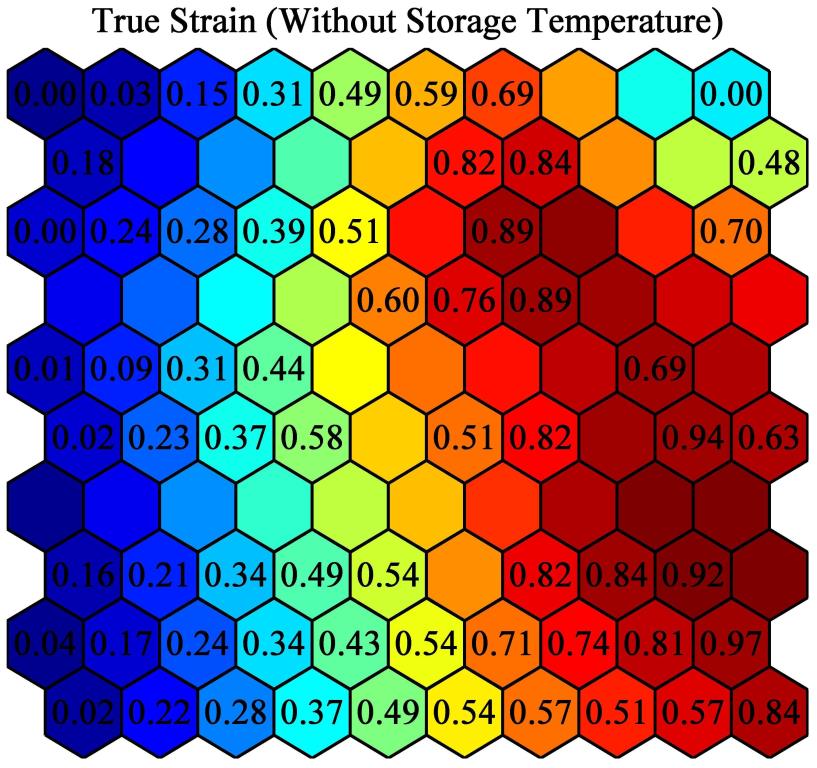

Supplement: Supplemental Material [file TBBE_A_1621206_SM3139.zip › Supplementary Figure 8(d).jpg]

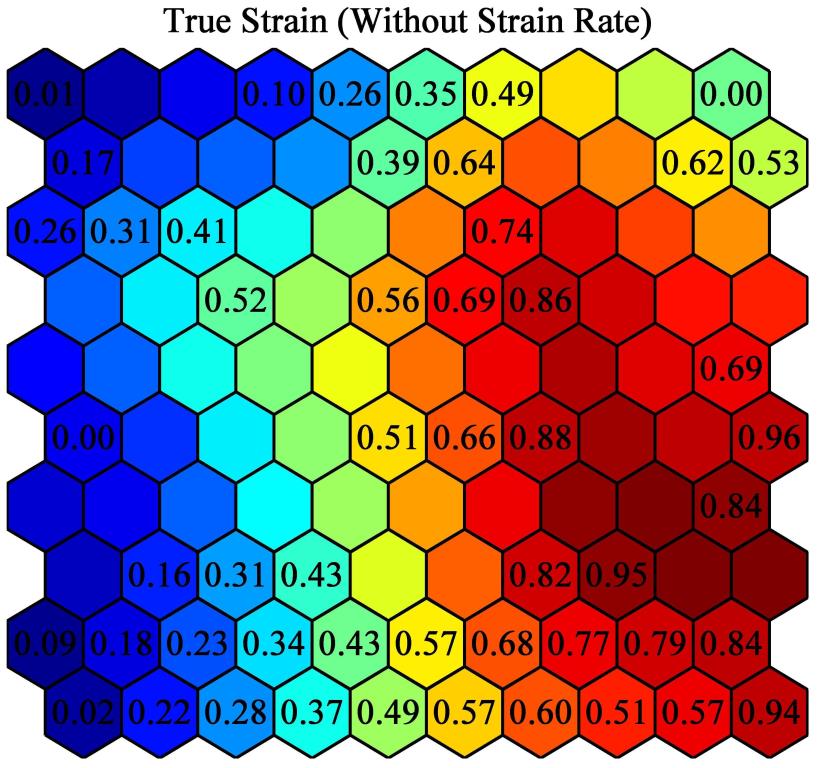

Supplement: Supplemental Material [file TBBE_A_1621206_SM3139.zip › Supplementary Figure 8(e).jpg]

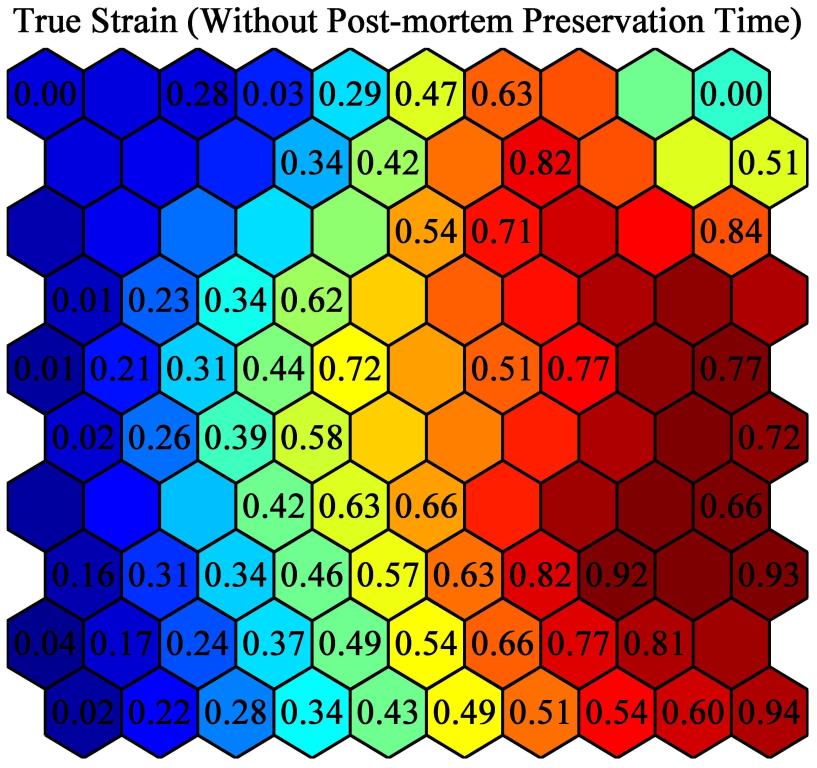

Supplement: Supplemental Material [file TBBE_A_1621206_SM3139.zip › Supplementary Figure 8(f).jpg]

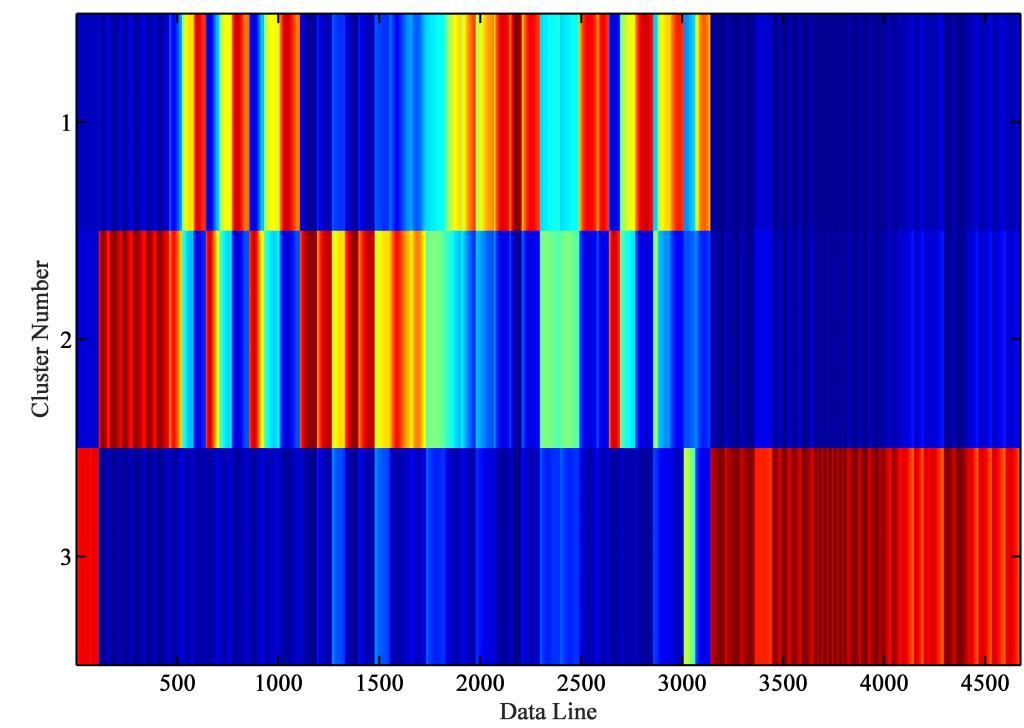

Supplement: Supplemental Material [file TBBE_A_1621206_SM3139.zip › Supplementary Figure 9.jpg]
